# Plastics-to-syngas photocatalysed by Co–Ga_2_O_3_ nanosheets

**DOI:** 10.1093/nsr/nwac011

**Published:** 2022-01-28

**Authors:** Jiaqi Xu, Xingchen Jiao, Kai Zheng, Weiwei Shao, Shan Zhu, Xiaodong Li, Junfa Zhu, Yang Pan, Yongfu Sun, Yi Xie

**Affiliations:** Hefei National Laboratory for Physical Sciences at Microscale, National Synchrotron Radiation Laboratory, University of Science and Technology of China, Hefei 230026, China; Key Laboratory of Green Chemistry & Technology, Ministry of Education, College of Chemistry, Sichuan University, Chengdu 610064, China; Hefei National Laboratory for Physical Sciences at Microscale, National Synchrotron Radiation Laboratory, University of Science and Technology of China, Hefei 230026, China; Hefei National Laboratory for Physical Sciences at Microscale, National Synchrotron Radiation Laboratory, University of Science and Technology of China, Hefei 230026, China; Hefei National Laboratory for Physical Sciences at Microscale, National Synchrotron Radiation Laboratory, University of Science and Technology of China, Hefei 230026, China; Hefei National Laboratory for Physical Sciences at Microscale, National Synchrotron Radiation Laboratory, University of Science and Technology of China, Hefei 230026, China; Hefei National Laboratory for Physical Sciences at Microscale, National Synchrotron Radiation Laboratory, University of Science and Technology of China, Hefei 230026, China; Hefei National Laboratory for Physical Sciences at Microscale, National Synchrotron Radiation Laboratory, University of Science and Technology of China, Hefei 230026, China; Hefei National Laboratory for Physical Sciences at Microscale, National Synchrotron Radiation Laboratory, University of Science and Technology of China, Hefei 230026, China; Hefei National Laboratory for Physical Sciences at Microscale, National Synchrotron Radiation Laboratory, University of Science and Technology of China, Hefei 230026, China; Institute of Energy, Hefei Comprehensive National Science Center, Hefei 230031, China; Hefei National Laboratory for Physical Sciences at Microscale, National Synchrotron Radiation Laboratory, University of Science and Technology of China, Hefei 230026, China; Institute of Energy, Hefei Comprehensive National Science Center, Hefei 230031, China

**Keywords:** commercial plastics, syngas, photoconversion, CO_2_ reduction, ambient conditions

## Abstract

Plastics take hundreds of years to degrade naturally, while their chemical degradation typically requires high temperature and pressure. Here, we first utilize solar energy to realize the sustainable and efficient plastic-to-syngas conversion with the aid of water at ambient conditions. As an example, the commercial plastic bags could be efficiently photoconverted into renewable syngas by Co–Ga_2_O_3_ nanosheets, with hydrogen and carbon monoxide formation rates of 647.8 and 158.3 μmol g^−1^ h^−1^. *In situ* characterizations and labelling experiments unveil water is photoreduced into hydrogen, while non-recyclable plastics including polyethylene bags, polypropylene boxes and polyethylene terephthalate bottles are photodegraded into carbon dioxide, which is further selectively photoreduced into carbon monoxide. In-depth investigation illustrates that the efficiency of syngas production mainly depends on the carbon dioxide reduction process and hence photocatalysts of high carbon dioxide reduction activity should be designed to promote the efficiency of plastic-to-syngas conversion in the future. The concept for the photoreforming of non-recyclable plastics into renewable syngas helps to eradicate ‘white pollution’ and alleviate the energy crisis simultaneously.

## Teaser

Non-recyclable commercial plastics are converted into syngas with solar as energy source in pure water at ambient conditions, which helps to alleviate ‘white pollution’ and the energy crisis simultaneously.

## INTRODUCTION

Plastic products, such as shopping bags, meal boxes and mineral-water bottles, have become one of the most widely used man-made materials in our daily life. It is estimated that ∼359 million tons of plastics are produced annually in the world and a total of ∼12 000 million metric tons of non-recyclable plastic wastes will have accumulated in the natural environment by 2050 [1]. Although these non-recyclable plastic wastes could spontaneously degrade over hundreds of years, they may also turn into microplastics that will invade water, plants and animals, and eventually transfer into the human body [2,3]. Recently, the World Health Organization announced that >90% of the prevailing bottled water that was tested contained more or less microplastics. Also, Mason *et al.* have reported that the microplastic concentrations of some bottled water could amount to >10 000 particles per liter [4]. More surprisingly, Schwabl *et al.* have confirmed that microplastics had for the first time been detected in human stools, implying unintentional ingestion of microplastics by humans [5]. All these studies suggest that non-recyclable plastic wastes in the environment are encroaching on human health. However, owing to their high stability and durability, it is still very difficult to rapidly degrade or sustainably recycle these discarded plastics [6,7]. Aside from the most widely used method of landfill, traditional strategies including pyrolysis and hydrocracking for the degradation of these non-recyclable plastic wastes usually require a high temperature up to 500°C [8]. Considering the abundant carbon sources of plastic wastes, they may serve as the raw material for the production of high-value-added fuels, for which many novel techniques have been employed to convert these non-recyclable plastic wastes into useful carbon-based compounds [9]. For example, Reisner *et al.* have realized the photoreforming of polyethylene terephthalate (PET) and polylactic acid (PLA) into hydrogen (H_2_) and some valuable carbon-based chemicals with the help of CN_x_|Ni_2_P catalysts and alkaline aqueous conditions [10], while they have also converted polyethylene (PE) into hydrocarbons through the integrated tandem chemical-photo/electrocatalytic processes [11]. Inspired by these great breakthroughs, it is a promising technology for utilizing solar energy to convert the non-recyclable plastic wastes into useful carbon-based compounds through a green and economic pathway.

In this regard, renewable syngas, primarily comprising carbon monoxide (CO) and H_2_, may be a promising target product since it could act as a versatile ingredient for preparing hydrocarbon fuels (methanol, ethanol, etc.) as well as hydrocarbon building blocks like ethylene and propylene [12]. To date, syngas is mainly produced by the reformation of fossil fuels, including coal, oil and natural gas, under rigorous and costly conditions (e.g. high temperature and pressure) [13,14]. For example, Ma *et al.* reported that the FeCralloy woven fiber-based catalyst could convert natural gas into CO and H_2_ at a temperature of 900°C and pressure of 0.1–2 MPa [15], while Zhou *et al.* demonstrated that the gasification of coal could produce CO, H_2_, methane (CH_4_) and carbon dioxide (CO_2_) at 700°C and 3.5 MPa [16]. Obviously, these strategies not only demand high energy consumption, but also result in a variety of complex by-products. In this case, the utilization of plastic wastes as feedstock for clean and renewable syngas production can contribute to the eradication of plastic pollution and the alleviation of a potential energy crisis simultaneously. Bearing in mind the discussion above, it is urgent to develop novel strategies for sustainably and efficiently converting non-recyclable plastic wastes into renewable syngas under mild conditions.

Herein, we first propose a sustainable and eco-friendly strategy for the photoreforming of non-recyclable plastics into clean renewable syngas with the assistance of H_2_O at ambient temperature and pressure. In this process, H_2_O could be photoreduced into H_2_, while various commercial plastics including PE bags, polypropylene (PP) boxes and PET bottles could be photodegraded into CO (Scheme S1). Compared with the time-consuming spontaneous degradation of plastics, the utilization of sunlight and suitable photocatalysts can help to realize the fast and sustainable conversion of plastics into renewable syngas, which could be further recycled to the plastics through the Fischer-Tropsch synthesis and polymerization processes.

## RESULTS AND DISCUSSION

Based on the aforementioned analysis, reasonable selection of highly active photocatalysts holds the key for realizing plastic degradation, CO_2_ reduction and H_2_O splitting to produce renewable syngas. Given this, earth-abundant and environmentally friendly Ga_2_O_3_ could be selected as a representative example to investigate the plastic-to-syngas conversion performance [17], since its valence-band (VB) maximum at approximately +3.16 V vs. NHE and conduction-band (CB) minimum at approximately −1.62 V vs. NHE at pH = 0 could satisfy some key redox potentials of CO_2_/CO (−0.1 V), H^+^/H_2_ (0 V) or O_2_/H_2_O (1.23 V), and even ·OH/H_2_O (2.73 V) that may take place in the photoconversion process [18,19]. However, the wide band gap of Ga_2_O_3_ may inversely result in lower photocatalytic performance owing to the poor solar energy utilization efficiency. In this regard, heteroatom doping can be utilized to tailor the electronic structure of Ga_2_O_3_, which could not only extend their photoabsorption, but also accelerate the carrier separation efficiency [20]. More importantly, it could provide a plentiful surface of exposed atoms by downsizing the materials into atomic thickness, which could act as highly active catalytic sites to boost the photoconversion property [21]. Accordingly, we employ Co-doped Ga_2_O_3_ (Co–Ga_2_O_3_) nanosheets and pristine Ga_2_O_3_ nanosheets as ideal models to explore the plastic-into-syngas process under mild conditions.

To this end, Co–Ga_2_O_3_ nanosheets were successfully fabricated for the first time. As displayed in Fig. S1, their XRD pattern could be indexed to γ-Ga_2_O_3_ (JCPDS card No. 19-4506). The transmission electron microscopy (TEM) image in Fig. S2 clearly reveals their ultra-thin morphology, while the high-resolution TEM image in Fig. S3A shows that the lattice plane spacings of Co–Ga_2_O_3_ nanosheets were 0.25 and 0.24 nm with a dihedral angle of 30°, in accordance with the calculated angle between the (113) and (222) planes of γ-Ga_2_O_3_, suggesting their [1 –1 0] orientation. The atomic force microscopy image in Fig. S3B and C indicates their average thickness of ∼1.05 nm, consisting well with the thickness of 12 atoms along the [1 –1 0] direction of γ-Ga_2_O_3_. More importantly, the annular dark-field TEM image and element mapping in Fig. S4 depict the homogeneous distribution of Ga, O and Co elements, while their X-ray photoelectron spectra of the Co 2p core level in Fig. S5 exhibits two feature satellite peaks at 787.1 and 804.1 eV, verifying the introduction of Co^2+^ in the synthetic sample [22]. For comparison, pristine Ga_2_O_3_ nanosheets with the same orientation and thickness were also fabricated by virtue of a similar synthetic strategy (Figs S1, S6 and S7). Furthermore, UV–vis diffuse reflectance spectra and synchrotron-radiation photoemission spectroscopy were utilized to unravel the electronic band structures for the Co–Ga_2_O_3_ nanosheets and the Ga_2_O_3_ nanosheets (Figs S8 and S9). Based on these results, it was demonstrated that the VB edge and the CB edge for the Co–Ga_2_O_3_ nanosheets were located at 2.50 and –1.40 V vs. NHE at pH = 7, while the VB and CB edges for the Ga_2_O_3_ nanosheets were located at 3.19 and –1.46 V vs. NHE at pH = 7. Thus, one can conclude that both of them are capable of realizing some key reactions such as H_2_O oxidation or CO_2_, O_2_ and H_2_O reduction [23,24].

**Figure 1.**
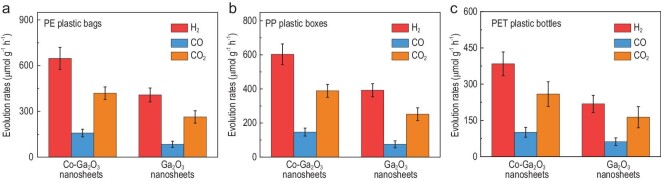
Efficient photoconversion of various plastics into syngas over Co–Ga_2_O_3_ nanosheets under mild conditions. Photoconversion of (a) commercial PE plastic bags, (b) commercial PP plastic boxes and (c) commercial PET plastic bottles under simulated AM 1.5G solar irradiation at ambient temperature and pressure: the formation rates of H_2_ (red), CO (blue) and CO_2_ (yellow) for Co–Ga_2_O_3_ nanosheets and Ga_2_O_3_ nanosheets in 24 h. The error bars in (a)–(c) represent the standard deviations of three independent measurements.

To disclose whether these two photocatalysts can realize the sustainable conversion of non-recyclable plastics into clean renewable syngas under mild conditions, the commonly used commercial plastic products of PE plastic bags, PP plastic boxes and PET plastic bottles were taken as the examples to perform the photocatalytic experiments, wherein the synthetic samples and the commercial plastics were mixed in pure water and irradiated by simulated sunlight (AM 1.5 G, 100 mW/cm^2^) at ambient temperature and pressure. Commercial plastic products of PE plastic bags, PP plastic boxes and PET plastic bottles were initially shredded into powders with a size of <5 mm using a pulverizer (Figs S10–S12). It should be mentioned that small plastics (≤5 mm) were commonly defined as the microplastics, which were particularly difficult to be recycled and may cause some unpredictable damage to the ecosystem. As displayed in Fig. 1a–c, Figs S13–S17 and Tables S1 and S2, the powders of commercial PE plastic bags, PP plastic boxes and PET plastic bottles could be efficiently photodegraded by both the Co–Ga_2_O_3_ nanosheets and the Ga_2_O_3_ nanosheets, in which the gas products of H_2_, CO and CO_2_ were detected by gas chromatography, while there was no detectable liquid product confirmed by the ^1^H NMR spectra in Fig. S13. Interestingly, there was plenty of CO_2_ dissolved in the water (Tables S5 and S6). It is worth noting that a handful of microplastics with smaller size were also detected (Fig. S14), which might have been produced during the photoconversion processes, conforming to the principle of carbon balance (please see the Methods section). Taking the commercial PE plastic bags as an example, the evolution rates of H_2_, CO and CO_2_ for the Co–Ga_2_O_3_ nanosheets were 647.8, 158.3 and 419.3 μmol g^–1^ h^–1^—roughly 1.6, 1.9 and 1.6 times higher than those of the Ga_2_O_3_ nanosheets, implying the former's better photoconversion performance. Note that the weight loss for the commercial PE plastic bags was 53% after 24 h irradiation over the Co–Ga_2_O_3_ nanosheets and the weight loss could reach 81% after 48 h irradiation (Fig. S15). More importantly, upon adding another 100 mg of commercial PE plastic bags to the photocatalytic system after 24 h irradiation, the Co–Ga_2_O_3_ nanosheets also possessed almost the same formation rates of H_2_, CO and CO_2_ (Fig. S16), suggesting their superb photocatalytic stability. Consequently, the commercial plastic products including PE plastic bags, PP plastic boxes and PET plastic bottles could be efficiently photodegraded into CO, while H_2_O could be photoreduced into H_2_ by the Co–Ga_2_O_3_ nanosheets.

**Figure 2.**
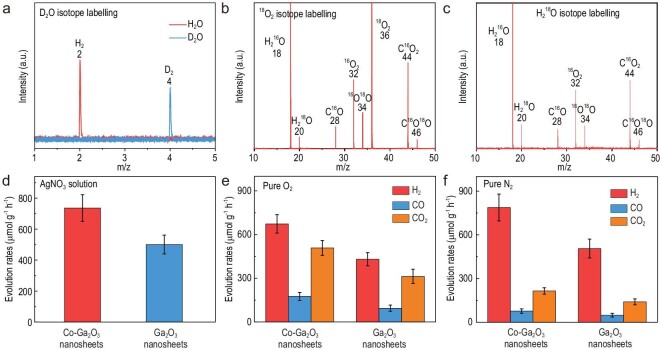
Investigation of mechanism involved in the photoconversion of reagent-grade PE into syngas over Co–Ga_2_O_3_ nanosheets. Synchrotron-radiation vacuum UV photoionization mass spectrometry (SVUV-PIMS) for the gas products in (a) the H_2_O and D_2_O isotope-labelling experiments after 24 h irradiation at hν = 16.5 eV; (b) ^18^O_2_ and (c) H_2_^18^O isotope-labelling experiments after 24 h irradiation at hν = 14.5 eV. (d) The formation rates of CO_2_ (no H_2_ or CO was detected) in 2 mmol/L AgNO_3_ solution, (e) the products in the pure O_2_ atmosphere, (f) the products in the pure N_2_ atmosphere. The error bars in (d)–(f) represent the standard deviations of three independent measurements.

To unveil the origin of the generated products, the photoreforming of reagent-grade PE was carried out over the Co–Ga_2_O_3_ nanosheets in an air atmosphere at ambient temperature and pressure with simulated sunlight. The evolution rates of H_2_, CO and CO_2_ were 692.0, 177.8 and 476.4 μmol g^–1^ h^–1^ (Fig. S17)—slightly higher than those for the commercial PE plastic bags. Of note, control experiments showed that H_2_ was detected, while there was no detectable CO and CO_2_ without reagent-grade PE in the photocatalytic system, suggesting that H_2_ may have come from the reduction of H_2_O (Table S3). To further disclose the origin of the H_2_, the photoreforming experiment of reagent-grade PE over the Co–Ga_2_O_3_ nanosheets was performed in the pure D_2_O solvent. Note that the synchrotron-radiation vacuum UV photoionization mass spectrometry (SVUV-PIMS) in Fig. 2a demonstrates that only D_2_ was detected in the D_2_O solvent, which firmly affirmed that the generated H_2_ originated from the H_2_O rather than the PE during the photoreforming processes. Moreover, when the light or photocatalyst or reagent-grade PE was removed, there was no detectable CO and CO_2_ (Table S3), verifying that CO and CO_2_ originated from the photodegradation of PE by the photocatalyst. Furthermore, H_2_ and O_2_ could be detected when no PE powders were added into the photocatalytic system under a N_2_ atmosphere (Fig. S18), implying that the H_2_O splitting could have been triggered by these two photocatalysts, in which some amount of H_2_O_2_ was also produced during the process (Fig. S19). To further testify the origin of the CO_2_ and CO, AgNO_3_ was added into the solution, which helped to clearly identify the photo-oxidation products since AgNO_3_ was usually considered as a trapping agent to efficiently consume the photo-generated electrons [25]; during the corresponding photocatalytic process, only CO_2_ was detected (Fig. 2d and Table S4), which certified that CO_2_ indeed derived from the photo-oxidation of PE. This result also indicated that CO may be generated from CO_2_ photoreduction, further attested to by the corresponding ^13^CO_2_ isotope-labelling experiments in Fig. S20. During the experiment of ^13^CO_2_ photoreduction in pure water, only ^13^CO was detected, which clearly affirmed that the generated CO stemmed from the reduction of CO_2_. Furthermore, when the reaction atmosphere changed from air to high-purity O_2_, the yields of CO and CO_2_ did not exhibit noticeable differences (Fig. 2e and Table S4). However, the yields of CO and CO_2_ for the Co–Ga_2_O_3_ nanosheets obviously decreased to 76.5 and 214.7 μmol g^–1^ h^–1^ in the high-purity N_2_ atmosphere (Fig. 2f and Table S4), hinting that O_2_ should be beneficial to the photoreforming processes. Additionally, the ^18^O_2_ isotope-labelling experiments in Fig. 2b clearly show the presence of C^16^O^18^O, demonstrating that O_2_ was indeed involved in the PE degradation processes. The identification of C^16^O^18^O in the H_2_^18^O labelling experiments (Fig. 2c) also implied the participation of H_2_O in the photodegradation of PE. Moreover, upon removing H_2_^18^O and ^18^O_2_ from the reaction system, there was no detectable C^16^O^18^O (Fig. S21), further confirming that H_2_O and O_2_ participated in the oxidation of PE into CO_2_. From the above-mentioned results, we deduce that the H_2_ stemmed from the H_2_O instead of the PE, while both the O_2_ and H_2_O took part in the oxidation of PE into CO_2_ and then the formed CO_2_ could be further reduced to CO.

**Figure 3.**
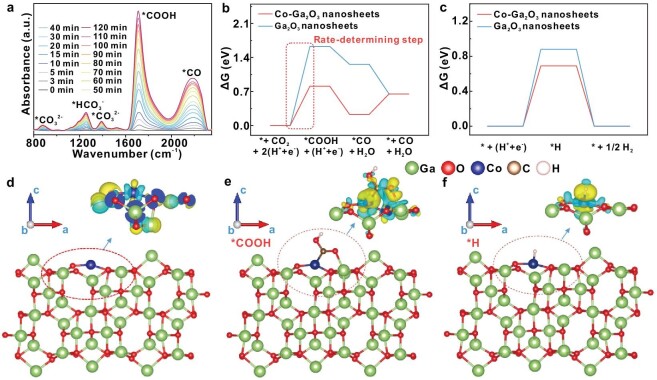
Investigation of Co atoms in the Co–Ga_2_O_3_ nanosheets for the plastic-to-syngas process. (a) *In situ* FT-IR spectra of Co–Ga_2_O_3_ nanosheets for the CO_2_ photoreduction process. Free-energy diagrams of (b) CO_2_ photoreduction to CO and (c) H_2_O photoreduction to H_2_ for the Co–Ga_2_O_3_ nanosheets and the Ga_2_O_3_ nanosheets. (d) The differential charge density map of the Co–Ga_2_O_3_ nanosheets. The differential charge density maps of (e) ^*^COOH and (f) H^*^ on the Co–Ga_2_O_3_ nanosheets. The yellow and blue contours in (d)–(f) manifest electron accumulation and depletion, and the values of isosurfaces in (d)–(f) are 0.007 and 0.002 eÅ^–3^, respectively.

To further understand the PE photoreforming processes, *in situ* electron spin resonance (ESR) spectra were employed to identify the reaction intermediates, where 5,5-dimethyl-1-pyrroline N-oxide (DMPO) was used as the spin-trapping agent. The ESR signals in Fig. S22 exhibit a 1 : 2 : 2 : 1 quartet pattern in the water solution, which could be assigned to the ·OH captured by the DMPO [26]. Combined with the H_2_^18^O isotope-labelling experiments in Fig. 2c, it is reasonable to deduce that H_2_O would be oxidized into ·OH radicals by the photo-induced holes and then the ·OH radicals would participate in the oxidation processes of the PE. Meanwhile, the ESR signals in Fig. S23 show a quartet pattern with an intensity of nearly 1 : 1 : 1 : 1 in the methanol solution, which could be assigned to the superoxide radicals (O_2_^•−^) captured by the DMPO [27]. As revealed in Fig. S24, the UV−vis absorption spectra for the reaction solution after 24 h irradiation displayed a characteristic peak at 436 nm, which could be ascribed to the characteristic peak of H_2_O_2_ [28]. Considering that O_2_ is in favor of the photodegradation of PE powders, it is possible that the O_2_ may undergo stepwise photoreduction into O_2_^•–^, H_2_O_2_ and H_2_O. Besides, the detection of C^16^O^18^O in both the H_2_^18^O and ^18^O_2_ labelling experiments suggested that both the ·OH radicals and the O_2_ participate in the photo-oxidation of PE into CO_2_. To gain in-depth investigation of the CO formation process, photocatalytic experiments in pure CO_2_ were conducted over the Co–Ga_2_O_3_ nanosheets, in which *in situ* FTIR spectra were performed to trace the reaction intermediates. As revealed in Fig. 3a, the new peak that appeared at ∼1710 and ∼2180 cm^–1^ under irradiation could be assigned to the ^*^COOH and ^*^CO groups, respectively [29,30]. It meant that CO_2_ molecules were initially reduced into COOH^*^ intermediates by the incoming proton–electron pair (H^+^ + e^–^); then, the COOH^*^ intermediates might have coupled with another H^+^ + e^–^ pair to form the ^*^CO intermediates, which would be liberated from the surface of the catalyst to form free CO molecules [31].

**Scheme 1.**
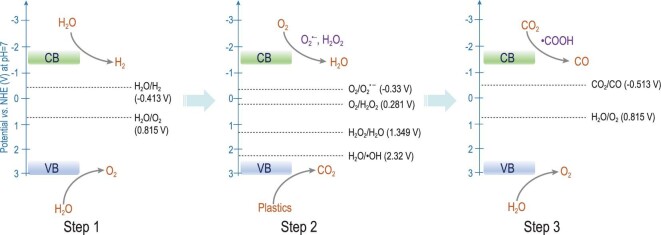
Schematic representation of the proposed mechanism for the photoconversion of non-recyclable plastics into renewable syngas under mild conditions.

From what has been mentioned above, it was concluded that the mechanism of photoconverting commercial plastics and H_2_O into syngas could be proposed as the following procedure. Under light irradiation, the photocatalysts initially generated electrons and holes pairs, which could react with the H_2_O to form H_2_ and O_2_. Then, the ·OH radicals derived from H_2_O and the produced O_2_ or O_2_ in the air atmosphere could synchronously photodegrade plastics into CO_2_; and meanwhile, O_2_ was also reduced to O_2_^•–^, H_2_O_2_ and H_2_O in order by the photoexcited electrons. Subsequently, the generated CO_2_ would be further reduced into CO through ^*^COOH intermediates, while H_2_O was oxidized into O_2_. In short, photoconverting commercial plastics and H_2_O into syngas may undergo the following three steps in sequence (Scheme 1):

Step 1: H_2_O was initially split into H_2_ and O_2_.

Step 2: Plastics were oxidized into CO_2_:

(1a) \begin{equation*} {\left( {{{\rm{C}}_2}{{\rm{H}}_4}} \right)_{\rm{n}}}\mathop {\longrightarrow} \limits^{\cdot{\rm{OH}},{{\rm{O}}_2}} 2{\rm{n}}\,{\rm{C}}{{\rm{O}}_2} + 2{\rm{n}}\,{{\rm{H}}_2}{\rm{O}}, \end{equation*}

(1b) \begin{equation*} {\left( {{{\rm{C}}_3}{{\rm{H}}_6}} \right)_{\rm{n}}}\mathop {\longrightarrow} \limits^{\cdot{\rm{OH}},\ {{\rm{O}}_2}} 3{\rm{n}}\,{\rm{C}}{{\rm{O}}_2} + 3{\rm{n}}\,{{\rm{H}}_2}{\rm{O}}, \end{equation*}

(1c) \begin{equation*} {\left( {{{\rm{C}}_{10}}{{\rm{H}}_8}{{\rm{O}}_4}} \right)_{\rm{n}}}\mathop {\longrightarrow} \limits^{\cdot{\rm{OH}},{{\rm{O}}_2}} 10{\rm{n}}\,\,{\rm{C}}{{\rm{O}}_2} + 4{\rm{n}}\,{{\rm{H}}_2}{\rm{O}}. \end{equation*}

Synchronously, O_2_ was reduced to H_2_O:

(2.1) \begin{equation*} {{\rm{O}}_2} + {{\rm{e}}^-} \to {{\rm{O}}_2}^{ \bullet -}, \end{equation*}

(2.2) \begin{equation*} {{\rm{O}}_2}^{ \bullet - } + {{\rm{e}}^ - } + 2{{\rm{H}}^ + } \to {{\rm{H}}_2}{{\rm{O}}_2}, \end{equation*}

(2.3) \begin{equation*} {{\rm{H}}_2}{{\rm{O}}_2} + 2{{\rm{e}}^ - } + 2{{\rm{H}}^ + } \to 2{{\rm{H}}_2}{\rm{O}}. \end{equation*}

Step 3: CO_2_ was further reduced to CO:

(3.1) \begin{equation*} {\rm{C}}{{\rm{O}}_2} + {{\rm{e}}^-} + {{\rm{H}}^ + } \to \cdot {\rm{COOH}}, \end{equation*}

(3.2) \begin{equation*} \cdot {\rm{COOH}} + {{\rm{e}}^-} + {{\rm{H}}^ + } \to {\rm{CO}} + {{\rm{H}}_2}{\rm{O}}. \end{equation*}

Meanwhile, H_2_O was oxidized to O_2_.

After disclosing the PE photoreforming processes, it is necessary to uncover the reasons for the boosted plastics photoconversion performance of Co–Ga_2_O_3_ nanosheets. Owing to the d–d internal transitions, the Co–Ga_2_O_3_ nanosheets possessed obviously enhanced photoabsorption relative to the Ga_2_O_3_ nanosheets (Fig. S8), which indicated that the former could make better use of the photon energy and generate more charge carriers to engage in the plastics photoreforming processes [18]. In addition, density-functional-theory calculations showed that the Co–Ga_2_O_3_ nanosheets possessed distinctly enhanced density of states at the VB edge (Fig. S25), which contributed to the promoted photocarrier transfer ability, and hence helped to provide more photo-generated electrons and holes to participate in the subsequent redox reactions [20]. Moreover, the photoluminescence (PL) intensity dramatically decreased after the introduction of Co atoms into the Ga_2_O_3_ nanosheets, indicating the restrained recombination efficiency of photo-generated electron–hole pairs (Fig. S26) [32]. Importantly, the CO_2_ temperature-programmed desorption spectra in Fig. S27 manifested that the onset desorption temperature of CO_2_ for the Co–Ga_2_O_3_ nanosheets (88°C) was higher than that of the Ga_2_O_3_ nanosheets (76°C), suggesting that the former has stronger adsorption behavior for CO_2_, which is beneficial to the further reduction of CO_2_ [33]. Furthermore, as uncovered by Fig. 3b and Tables S7 and S8, the calculated reaction Gibbs free energies (ΔG) showed that the rate-determining step of CO_2_ photoreduction into CO was the formation of ^*^COOH intermediates, wherein the Co–Ga_2_O_3_ nanosheets possessed a lower COOH^*^ intermediates formation energy compared with the Ga_2_O_3_ nanosheets. This could be attributed to the fact that the introduction of Co atoms caused the charge accumulation of the Co atoms and the neighboring Ga atoms, which was conducive to stabilizing the ^*^COOH intermediates through Co–Ga dual active sites and hence lowered its formation energy (Fig. 3d and e, and Figs S28 and S29) [34]. More importantly, the calculated reaction Gibbs free energies in Fig. 3c and Fig. S30 disclose that the rate-determining step of H_2_O reduction into H_2_ was the formation of H^*^ intermediates, in which the Co–Ga_2_O_3_ nanosheets possessed a lower H^*^ intermediates formation energy with respect to the Ga_2_O_3_ nanosheets. This might be ascribed to the charge accumulation of the Co–H bond induced by the introduced Co atoms, which helped to stabilize the H^*^ intermediates, thus lowering the formation energy (Fig. 3f). As a result, both theoretical and experimental results affirmed that introducing Co atoms into Ga_2_O_3_ nanosheets as the active sites can simultaneously favor the processes of CO_2_ reduction into CO and H_2_O reduction into H_2_ through stabilizing the ^*^COOH and H^*^ intermediates. That is to say, the presence of Co atoms could make for lowing the activation energies of CO_2_ reduction and H_2_O splitting, and hence promote the property of plastic-to-syngas photoconversion.

## CONCLUSION

In conclusion, we first realized the sustainable and efficient conversion of non-recyclable plastics into renewable syngas in pure water at ambient temperature and pressure. In this process, H_2_O is photoreduced into H_2_, while non-recyclable plastics including PE plastic bags, PP plastic boxes and PET plastic bottles are photodegraded into CO_2_, which is further selectively photoreduced into CO. As an example, commercial PE plastic bags could be efficiently photoconverted into syngas with the aid of H_2_O by the Co–Ga_2_O_3_ nanosheets, in which the H_2_ and CO formation rates were ∼647.8 and ∼158.3 μmol g^−1^ h^−1^ —roughly 1.6 and 1.9 times higher than those of the Ga_2_O_3_ nanosheets, respectively. More importantly, the weight losses of PE plastic bags, PP plastic boxes and PET plastic bottles were up to approximately 81%, 78% and 72% after 48 h irradiation over the Co–Ga_2_O_3_ nanosheets. Through deeply exploring the mechanism of plastics photoconversion into syngas, it can be concluded that the whole efficiency is mainly dependent on the process of CO_2_ reduction into CO, and hence it is necessary to design photocatalysts of high CO_2_ reduction activity to promote the efficiency of non-recyclable plastics degradation in the future. Meanwhile, considering that the residual microplastics in aqueous solution are hard to fully degrade, some precautionary measures such as filtration units and advanced wastewater treatment technologies like membrane filtration may be needed in the future. Briefly, the design concept may help to open up new avenues toward the curbing of white pollution and the relieving of the energy crisis simultaneously.

## METHODS

### Synthesis of the Co-doped Ga_2_O_3_ (Co–Ga_2_O_3_) nanosheets

In a typical synthetic procedure, 256 mg Ga(NO_3_)_3_ and 29 mg Co(NO_3_)_2_ · 6H_2_O were added to 5 mL water with vigorous stirring for 10 min. Then, 30 mL triethylenetetramine (TETA) was added to the solution after continuous magnetic stirring for another 30 min. Finally, the solution was transferred to a stainless Teflon-lined autoclave, which was sealed and maintained at 160°C for 48 h. The product was collected by centrifugation, washed thoroughly with absolute ethanol and deionized water several times, and then dried at 60°C for 12 h.

### Synthesis of the Ga_2_O_3_ nanosheets

The procedures were the same as those for the Co–Ga_2_O_3_ nanosheets, except no Co(NO_3_)_2_ · 6H_2_O was added.

## Supplementary Material

nwac011_Supplemental_File

## References

[1] Geyer R , Jambeck JR, Law KL. Production, use, and fate of all plastics ever made. Sci Adv 2017; 3: e1700782. 10.1126/sciadv.1700782 28776036 PMC5517107

[2] Seltenrich N . New link in the food chain? Marine plastic pollution and seafood safety. Environ Health Perspect 2015; 123: A34. 10.1289/ehp.123-A34 25643424 PMC4314237

[3] Rochman CM , Hoh E, Kurobe T et al. Ingested plastic transfers hazardous chemicals to fish and induces hepatic stress. Sci Rep 2013; 3: 3263. 10.1038/srep03263 24263561 PMC3836290

[4] Mason SA , Welch VG, Neratko J. Synthetic polymer contamination in bottled water. Front Chem 2018; 6: 407. 10.3389/fchem.2018.00407 30255015 PMC6141690

[5] Schwabl P , Koppel S, Konigshofer P et al. Detection of various microplastics in human stool a prospective case series. Ann Intern Med 2019; 171: 453–7. 10.7326/M19-0618 31476765

[6] Tournier V , Topham CM, Gilles A et al. An engineered PET depolymerase to break down and recycle plastic bottles. Nature 2020; 580: 216–9. 10.1038/s41586-020-2149-4 32269349

[7] Garcia JM , Robertson ML. The future of plastics recycling chemical advances are increasing the proportion of polymer waste that can be recycled. Science 2017; 358: 870–2. 10.1126/science.aaq0324 29146798

[8] Nisar J , Ali M, Awan IA. Catalytic thermal decomposition of polyethylene by pyrolysis gas chromatography. J Chil Chem Soc 2011; 56: 653–5. 10.4067/S0717-97072011000200006

[9] Estahbanati MR , Kong XY, Eslami A et al. Current developments in the chemical upcycling of waste plastics using alternative energy sources. ChemSusChem 2021; 14: 4152–66. 10.1002/cssc.202100874 34048150

[10] Uekert T , Kasap H, Reisner E et al. Photoreforming of nonrecyclable plastic waste over a carbon nitride/nickel phosphide catalyst. J Am Chem Soc 2019; 141: 15201–10. 10.1021/jacs.9b06872 31462034 PMC7007225

[11] Pichler CM , Bhattacharjee S, Rahaman M et al. Conversion of polyethylene waste into gaseous hydrocarbons via integrated tandem chemical-photo/electrocatalytic processes. ACS Catal 2021; 11: 9159–67. 10.1021/acscatal.1c02133 34386271 PMC8353629

[12] Nguyen VN , Blum L. Syngas and synfuels from H_2_O and CO_2_: current status. Chem Ing Tech 2015; 87: 354–75. 10.1002/cite.201400090

[13] Chen LW , Gangadharan P, Lou HH. Sustainability assessment of combined steam and dry reforming versus tri-reforming of methane for syngas production. Asia-Pac J Chem Eng 2018; 13: e2168. 10.1002/apj.2168

[14] Gur M , Canbaz ED. Analysis of syngas production and reaction zones in hydrogen oriented underground coal gasification. Fuel 2020; 269: 117331. 10.1016/j.fuel.2020.117331

[15] Ma ZN , Ouzilleau P, Trevisanut C et al. Partial oxidation of methane to syngas over Pt/Rh/MgO catalyst supported on FeCralloy woven fibre. Can J Chem Eng 2016; 94: 642–9. 10.1002/cjce.22428

[16] Zhou X , Zhao J, Guo S et al. High quality syngas production from pressurized K_2_CO_3_ catalytic coal gasification with in-situ CO_2_ capture. Int J Hydrogen Energy 2018; 43: 17091–9. 10.1016/j.ijhydene.2018.07.062

[17] Sun H , Zhang L, Yu J et al. Phase junction enhanced photocatalytic activity of Ga_2_O_3_ nanorod arrays on flexible glass fiber fabric. RSC Adv 2020; 10: 11499–506. 10.1039/D0RA01461C 35495304 PMC9050499

[18] Chang XX , Wang T, Gong JL. CO_2_ photo-reduction: insights into CO_2_ activation and reaction on surfaces of photocatalysts. Energy Environ Sci 2016; 9: 2177–96. 10.1039/C6EE00383D

[19] Navarrete M , Cipagauta DS, Gómez R. Ga_2_O_3_/TiO_2_ semiconductors free of noble metals for the photocatalytic hydrogen production in a water/methanol mixture. J Chem Technol Biotechnol 2019; 94: 3457–65. 10.1002/jctb.5967

[20] Kikkawa S , Teramura K, Asakura H et al. Development of Rh-doped Ga_2_O_3_ photocatalysts for reduction of CO_2_ by H_2_O as an electron donor at a more than 300 nm wavelength. J Phys Chem C 2018; 122: 21132–9. 10.1021/acs.jpcc.8b04956

[21] Sun Z , Talreja N, Tao H et al. Catalysis of carbon dioxide photoreduction on nanosheets: fundamentals and challenges. Angew Chem Int Ed 2018; 57: 7610–27. 10.1002/anie.201710509 29219235

[22] Xu J , Li X, Ju Z et al. Visible-light-driven overall water splitting boosted by tetrahedrally coordinated blende cobalt(II) oxide nanosheets. Angew Chem Int Ed 2019; 58: 3032–6. 10.1002/anie.201807332 30137662

[23] Wood PM . The potential diagram for oxygen at pH 7. Biochem J 1988; 253: 287–9. 10.1042/bj2530287 2844170 PMC1149288

[24] Sun Z , Ma T, Tao H et al. Fundamentals and challenges of electrochemical CO_2_ reduction using two-dimensional materials. Chem 2017; 3: 560–87. 10.1016/j.chempr.2017.09.009

[25] Allen MR , Thibert A, Sabio EM et al. Evolution of physical and photocatalytic properties in the layered titanates A_2_Ti_4_O_9_ (A = K, H) and in nanosheets derived by chemical exfoliation. Chem Mater 2009; 22: 1220–8. 10.1021/cm902695r

[26] Ho W , Jimmy CY, Lee S. Synthesis of hierarchical nanoporous F-doped TiO_2_ spheres with visible light photocatalytic activity. Chem Commun 2006: 1115–7. 10.1039/b515513d 16514457

[27] Zhang N , Li X, Ye H et al. Oxide defect engineering enables to couple solar energy into oxygen activation. J Am Chem Soc 2016; 138: 8928–35. 10.1021/jacs.6b04629 27351805

[28] Guo F , Shi W, Zhu C et al. CoO and g-C_3_N_4_ complement each other for highly efficient overall water splitting under visible light. Appl Catal B 2018; 226: 412–20. 10.1016/j.apcatb.2017.12.064

[29] Giotta L , Mastrogiacomo D, Italiano F et al. Reversible binding of metal ions onto bacterial layers revealed by protonation-induced ATR-FTIR difference spectroscopy. Langmuir 2011; 27: 3762–73. 10.1021/la104868m 21395289

[30] Zeng L , Li K, Wang H et al. CO oxidation on Au/alpha-Fe_2_O_3_-hollow catalysts: general synthesis and structural dependence. J Phys Chem C 2017; 121: 12696–710. 10.1021/acs.jpcc.7b01363

[31] Xu JQ , Li XD, Liu W et al. Carbon dioxide electroreduction into syngas boosted by a partially delocalized charge in molybdenum sulfide selenide alloy monolayers. Angew Chem Int Ed 2017; 56: 9121–5. 10.1002/anie.201704928 28621043

[32] Wu J , Li XD, Shi W et al. Efficient visible-light-driven CO_2_ reduction mediated by defect-engineered BiOBr nanosheets. Angew Chem Int Ed 2018; 57: 8719–23. 10.1002/anie.201803514 29761617

[33] Jiao XC , Li XD, Jin XY et al. Partially oxidized SnS_2_ nanosheets achieving efficient visible-light-driven CO_2_ reduction. J Am Chem Soc 2017; 139: 18044–51. 10.1021/jacs.7b10287 29144744

[34] Li XD , Sun YF, Xu JQ et al. Selective visible-light-driven photocatalytic CO_2_ reduction to CH_4_ mediated by atomically thin CuIn_5_S_8_ layers. Nat Energy 2019; 4: 690–9. 10.1038/s41560-019-0431-1

