# Supplementary material for: Plastics-to-syngas photocatalysed by Co–Ga2O3 nanosheets
Source: Natl Sci Rev. 2022 Jan 28;9(9):nwac011. doi: 10.1093/nsr/nwac011 (PMC9564184; doi:10.1093/nsr/nwac011)
Supplement: nwac011_Supplemental_File [file nwac011_supplemental_file.pdf]

Supplementary Information

Plastics-to-Syngas Photocatalyzed by Co-Ga_2O_3 Nanosheets

Jiaqi Xu,[1,3,+] Xingchen Jiao,[1,+] Kai Zheng,[1,+] Weiwei Shao,[1] Shan Zhu,[1] Xiaodong Li,[1] Junfa Zhu,[1] Yang Pan,[1] Yongfu Sun,[1,2]* and Yi Xie[1,2]

[1]Hefei National Laboratory for Physical Sciences at Microscale, National Synchrotron Radiation Laboratory, University of Science and Technology of China, Hefei 230026, China.

[2]Institute of Energy, Hefei Comprehensive National Science Center, Hefei, Anhui 230031, China.

[3]Key Laboratory of Green Chemistry & Technology, Ministry of Education, College of Chemistry, Sichuan University, Chengdu 610064, China.

[+]These authors contributed equally to this work.

Email: yfsun@ustc.edu.cn

EXPERIMENTAL SECTION

Characterization

Transmission electron microscopy (TEM) images were obtained from a JEOL-2010 TEM. High-resolution TEM (HRTEM) images and the corresponding elemental mapping images were measured on a JEOL JEM-ARM200F TEM/STEM. The field-emission scanning electron microscopy (FE-SEM) images were performed by using a FEI Sirion-200 SEM. X-ray diffraction (XRD) patterns were recorded by a Philips X'Pert Pro Super diffractometer with Cu Kα radiation (λ = 1.54178 Å), in which the voltage was 40 kV, the electric current was 100 mA and the scan speed was 35 °/min. Atomic force microscopy (AFM) was carried out on a Veeco DI Nano-scope multimode V system with a scan speed of 1.00 Hz. X-ray photoelectron spectra (XPS) were measured on an ESCALAB MKII with Al Kα (hυ = 1486.6 eV) as the excitation source. The binding energies obtained in the XPS spectral analysis were corrected by referencing C 1s to 284.8 eV. UV−vis diffuse reflectance spectra were performed on a Perkin Elmer Lambda 950 UV−vis−NIR spectrophotometerand the UV−vis absorption spectrum was recorded on UV-2501PC/2550 (Shimadzu Corporation, Japan). Room temperature photoluminescence (PL) spectra were evaluated by a LabRAM HR Evolution Micro-Raman system, the excitation wavelength was 325 nm, and the PL spectra were accumulated for 30 s with an output power of ca. 1.7 mW. The CO_2 temperature programmed desorption (TPD) measurements were carried out on AutoChem II 2920 with a temperature ramp rare of 10 ℃/min. *In situ* FT-IR spectra were executed by a Thermo Scientific Nicolet iS50. *In situ* ESR spectra were obtained using a JEOL JESFA200 ESR spectrometer. Synchrotron radiation photoemission spectroscopy (SRPES) was performed at the Catalysis and Surface Science Endstation at the BL11U beamline in the National Synchrotron Radiation Laboratory (NSRL) in Hefei, China. The work function (Φ) is determined by the difference between the photon energy and the binding energy of the secondary cutoff edge, according to the formula of $\Phi = h\upsilon - E_{cutoff}$ (hυ refers to the utilized photon energy of 40.00 eV and a sample bias of −5 V was applied to observe the secondary electron cutoff).

Photoconversion of Commercial Plastic Products and Reagent-Grade PE Powders

Commercial plastic products of PE plastic bags, PP plastic boxes and PET plastic bottles were initially crushed into fine powders (<5 mm) by a pulverizer (Benchen Science and Technology, Figures S10-S12). Reagent-grade PE powders with the size of ca. 5 mm were purchased from Alfa Aesar (China) without any further purification. It should be mentioned that small plastics (≤5 mm) were commonly defined as the microplastics, which were particularly difficult to be recycled and may cause some unpredictable damages to the ecosystem. Then, a closed glass gas-circulation system (Labsolar-IIIAG, Beijing

Perfectlight Technology Co., Ltd) was used to conduct the photoconversion tests. In detail, 50 mg photocatalysts and 100 mg plastic products powders were initially dispersed in 100 mL deionized water under stirring. The dispersion was put into the reaction vessel, which was soaked in an outer vessel so that cooling water could circulate to keep the temperature of the reaction solution at 298 $\pm$ 0.2 K. The reaction system was vacuum-treated and then filled with high-purity air to reach an atmospheric pressure. The light source for the photocatalysis was a 300 W Xe lamp (CEL-HXFUV300, Beijing Ceaulight Technology Co., Ltd., China) with a standard AM 1.5 filter to simulate one-sun irradiation, in which the outputting light density was about 100 mW/cm^2. During the light irradiation, the evolved gas products were qualitatively and quantificationally analyzed by Agilent GC-7890B gas chromatograph. The liquid products were quantified by NMR (Bruker AVANCE AV III 400) spectroscopy, in which 0.4 mL supernate was mixed with 0.1 mL D_2O (deuterated water) and 0.02 mL dimethyl sulfoxide (DMSO, Sigma, 99.99%) was added as an internal standard. The one-dimensional ^{1}H spectrum was measured with water suppression using a pre-saturation method. To analyze the content of CO_2 dissolved in the solution, 2.0 mL supernate was taken immediately after the photocatalysis. Upon adding excess $Ba(OH)_2$ powders to the solution, the white precipitate could be obtained. Taking the Co-Ga_2O_3 nanosheets as examples, after the following centrifugation and the gravimetric analysis for the white precipitate, the produced CO_2 and CO dissolved in the solution after the photoconversion of pure PE or PP or PET could be calculated to be ca. 2.88, 2.53 and 1.65 mmol, respectively. And meanwhile, the amount of gas CO_2 and CO after the photoconversion of pure PE or PP or PET detected by gas chromatograph was roughly 0.69, 0.64 and 0.43 mmol, respectively. That is to say, the total carbon content for the photoconversion of all these three plastics over both the two catalysts can exceed 90% (Table S5-6). In addition, a handful of microplastics with smaller size were also detected by the SEM images (Fig. S14), which might be produced during the photoconversion processes, conforming to the principle of carbon balance.

Detection of the Isotope Tracing Gas Products

Synchrotron-based vacuum ultraviolet photoionization mass spectrometry (SVUV-PIMS) was carried out at the combustion endstation at the BL03U beamline at the National Synchrotron Radiation Laboratory at Hefei, China. In detail, 50 mg Co-Ga_2O_3 nanosheets and 100 mg reagent-grade PE powders were initially dispersed in 100 mL deionized water under stirring. The reaction system was vacuum-treated three times to remove air completely, which was then pumped by high-purity N_2 to reach an atmospheric pressure. For the ^{18}O or $H_2{}^{18}O$ labelling experiments, a small amount of $^{18}O_2$ or 1 mL $H_2{}^{18}O$ were added to the reaction system, and a certain amount of gas was taken immediately after 24 h irradiation and was analyzed at the photon energy of 14.5 eV. For the D_2 labelling experiments, D_2O were

utilized to replace H_2O.

In situ FTIR experiments

All FTIR spectra were recorded on Thermo Scientific Nicolet iS50. The spectra were displayed in absorbance units and acquired with a MCT detector and a resolution of 8 cm^{-1}, using 64 scans. The dome of the reaction cell had two KBr windows allowing IR transmission and a third window allowing transmission of irradiation introduced through a liquid light guide that connects to a 300 W Xe lamp. The Co-Ga_2O_3 nanosheets and Ga_2O_3 nanosheets powders were added to the reaction cell. After spraying some water on the powders, the reaction cell was sealed. Then, the reaction system was vacuum-treated three times to remove air completely, which was then pumped by high-purity CO_2 to reach an atmospheric pressure. Next, the FTIR spectra were recorded as a function of time to investigate the dynamics of the reactant adsorption in the dark and desorption/conversion under irradiation.

In situ ESR Experiments

(1) Detection of superoxide radical anion ($O_2^{\cdot-}$): 5 mg Co-Ga_2O_3 nanosheets and 15 mg reagent-grade PE powders were dispersed in 1 mL of methanol followed by addition of 20 μL DMPO methanol solution (100 mM). After 10 s irradiation, the mixed reaction solution was analyzed by in situ ESR spectra.

(2) Detection of hydroxyl radical (·OH): 5 mg Co-Ga_2O_3 nanosheets and 15 mg reagent-grade PE powders were dispersed in 1 mL of H_2O followed by addition of 1 mL DMPO solution (2 mM in water). After 10 s irradiation, the mixed reaction solution was analyzed by in situ ESR spectra.

Detection of H_2O_2

2.0 mL reaction solution was taken immediately after 24 h photoconversion of reagent-grade PE powders. Then, 2 mL 1% o-tolidine in 0.1 M HCl was added to the above reaction solution. Subsequently, the solution was acidified with 1 M HCl (2 mL). The absorption spectrum of the solution was immediately recorded with a UV–vis spectrophotometer and the H_2O_2 has a characteristic absorption peak at ca. 436 nm.

Detection of H_2O_2 with o-tolidine

Hydrogen peroxide (H_2O_2) measurements were performed on an UV−vis−NIR spectrophotometer. 2.0 mL reaction solution was taken immediately after 5 h irradiation of pure PE over the Co-Ga_2O_3 nanosheets under simulated natural environments. Then, 2 mL 1% o-tolidine in 0.1 M HCl was added to the above reaction solution. Subsequently, the solution was acidified with 1 M HCl (2 mL). The absorption spectrum

of the solution was immediately recorded with a UV–vis spectrophotometer. The H_2O_2 has a characteristic absorption peak at 436 nm.

CO_2 Photoreduction Tests

A closed glass gas-circulation system (Labsolar-IIIAG, Beijing Perfectlight Technology Co., Ltd) was used to conduct the photocatalytic CO_2 tests. In detail, 50 mg Co-Ga_2O_3 nanosheets or Ga_2O_3 nanosheets were initially dispersed in 100 mL deionized water under stirring. The dispersion was put into the reaction vessel, which was soaked in an outer vessel so that cooling water could circulate to keep the temperature of the reaction solution at room temperature. Then, the reaction system was vacuum-treated three times to remove air completely, which was then pumped by high-purity CO_2 ($^{13}CO_2$ was used for the ^{13}C labelling experiments) to reach an atmospheric pressure. The light source for the photocatalysis was a 300 W Xe lamp (CEL-HXFUV300, Beijing Ceaulight Technology Co., Ltd., China) with a standard AM 1.5 filter, in which the outputting light density was about 100 mW/cm^2.

Calculation Details

The first-principles calculations were performed with the Vienna ab initio simulation package.[1,2] The interaction between ions and valence electrons was described using projector augmented wave (PAW) potentials, and the exchange–correlation between electrons was treated through using the generalized gradient approximation (GGA) in the Perdew–Burke–Ernzerhof (PBE) form.[3] To achieve the accurate density of the electronic states, the plane wave cutoff energy was 480 eV, a gamma-centre 3 × 9 × 1 for sheet k-point mesh were used. Ionic relaxations were carried out under the conventional energy (10^{-5} eV) and force (0.01 eV/Å) convergence criteria. All periodic slabs have a vacuum spacing of 15 Å to avoid the interaction between adjacent layers. The structure model of Co-Ga_2O_3 nanosheets and Ga_2O_3 nanosheets contain a supercell size of a = 17.37 Å, b = 5.96 Å, c = 25.66 Å, α = β = γ = 90º.

Gibbs free energies for each gaseous and adsorbed species were calculated at 298.15 K, according to the expression:

$$G = E_{DFT} + E_{ZPE} - TS \quad \text{(Eq. S1)}$$

$$E_{ZPE} = \sum_i 1/2\, h\nu_i \quad \text{(Eq. S2)}$$

$$\Theta_i = h\nu_i / k \quad \text{(Eq. S3)}$$

$$S = \sum_i R[\ln(1 - e^{-\Theta_i/T})^{-1} + \frac{\Theta_i}{T}(e^{\Theta_i/T} - 1)^{-1}] \quad \text{(Eq. S4)}$$

Where E_{DFT} is the electronic energy calculated for specified geometrical structures, E_{ZPE} is the zero-point energy, S is the entropy, h is the Planck constant, ν is the computed vibrational frequencies, Θ is the

characteristic temperature of vibration, k is the Boltzmann constant, and R is the molar gas constant. For adsorbates, all 3N degrees of freedom were treated as frustrated harmonic vibrations with negligible contributions from the catalysts' surfaces. No bias was applied (U = 0 V), the free energy of the (H^{+}+e^{-}) pair is related to that of 1/2 H_2 in the gas-phase. Free energy changes with respect to an initial state of gaseous CO_2 and H_2 freely above an empty surface can be represented by the following:

$$\Delta G[COOH^*] = G[COOH^*] + G[H^+ + e^-] - (G[^*] + G[CO_2] + 2\times G[H^+ + e^-]) \quad \text{(Eq. S5)}$$

$$\Delta G[CO^*] = G[CO^*] + G[H_2O] - (G[^*] + G[CO_2] + 2\times G[H^+ + e^-]) \quad \text{(Eq. S6)}$$

$$\Delta G[H^*] = G[H^*] - (G[^*] + G[H^+ + e^-]) \quad \text{(Eq. S7)}$$

$$G[H^+ + e^-] = 1/2G[H_2] \quad \text{(Eq. S8)}$$

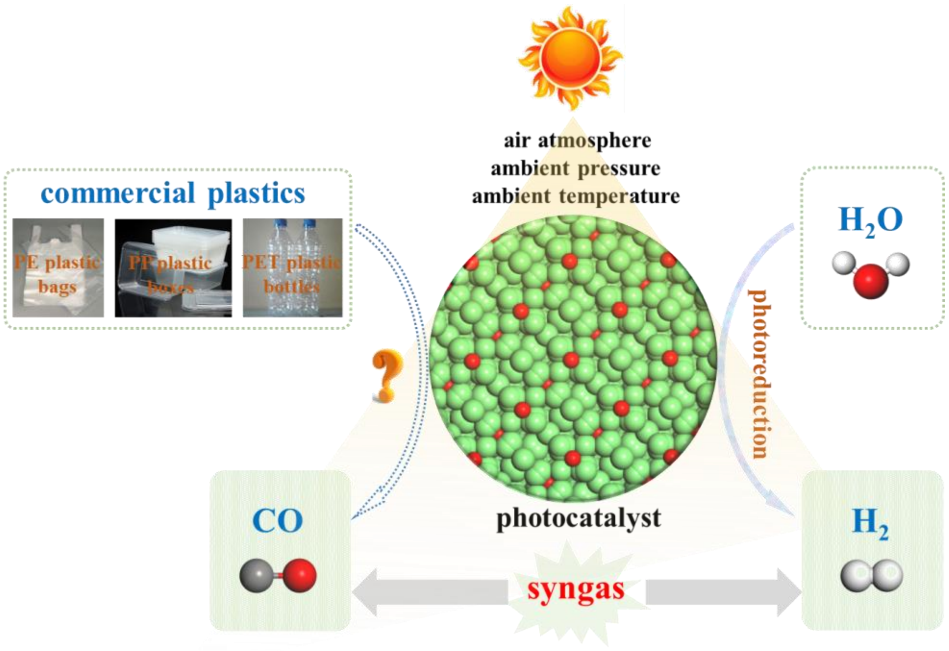

Scheme S1. Schematic representations for the photoconversion of non-recyclable plastics into syngas. In the photoconversion process, various commercial plastics and H_2O were sustainably and efficiently converting into renewable syngas under mild conditions (25 °C, 1 atm). That is to say, commercial plastics are photodegraded to CO, while H_2O is photoreduced to H_2 over the same photocatalyst.

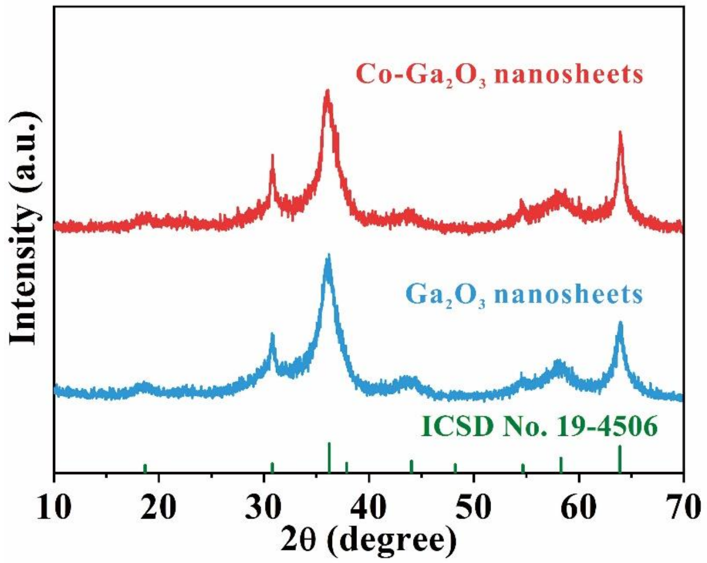

Figure S1 XRD patterns of the Co-Ga_2O_3 nanosheets and the Ga_2O_3 nanosheets.

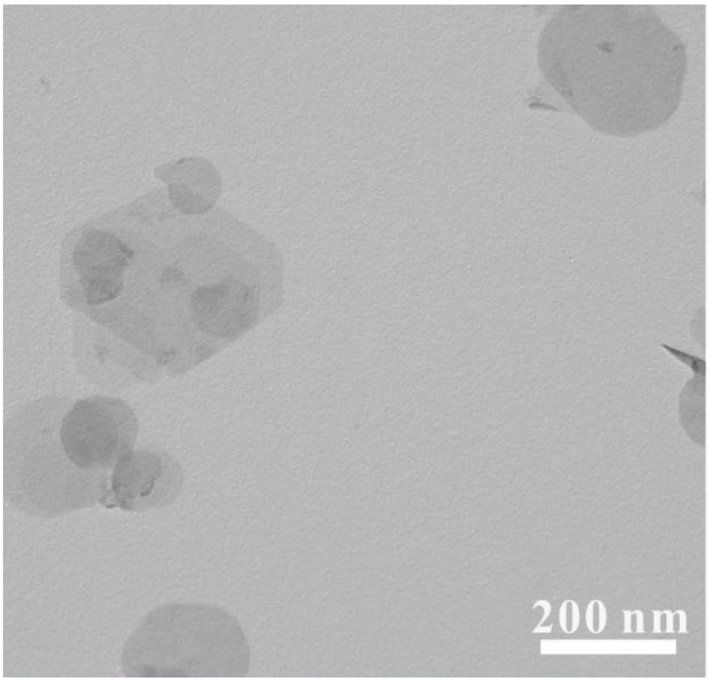

Figure S2 TEM image of the Co-Ga_2O_3 nanosheets.

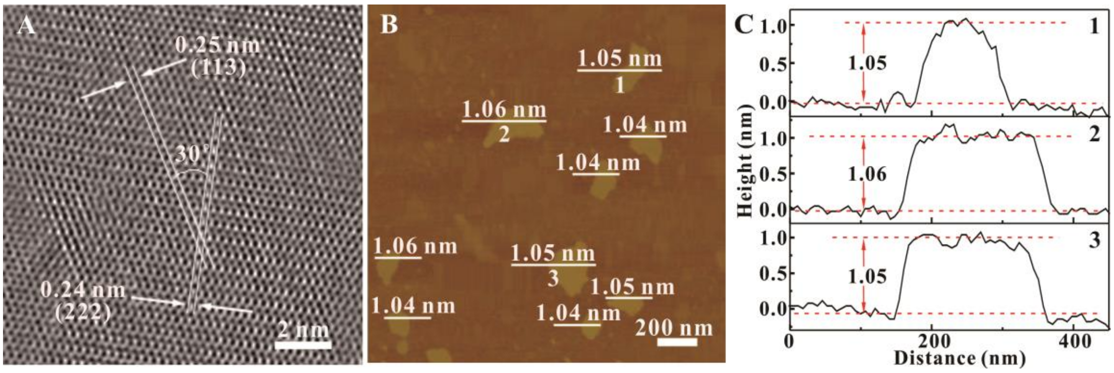

Figure S3 (A) High-resolution TEM image, (B) AFM image and (C) the corresponding height profiles of the Co-Ga_2O_3 nanosheets. The number 1, 2 and 3 in (B) correspond to the numbers 1 to 3 in (C), respectively.

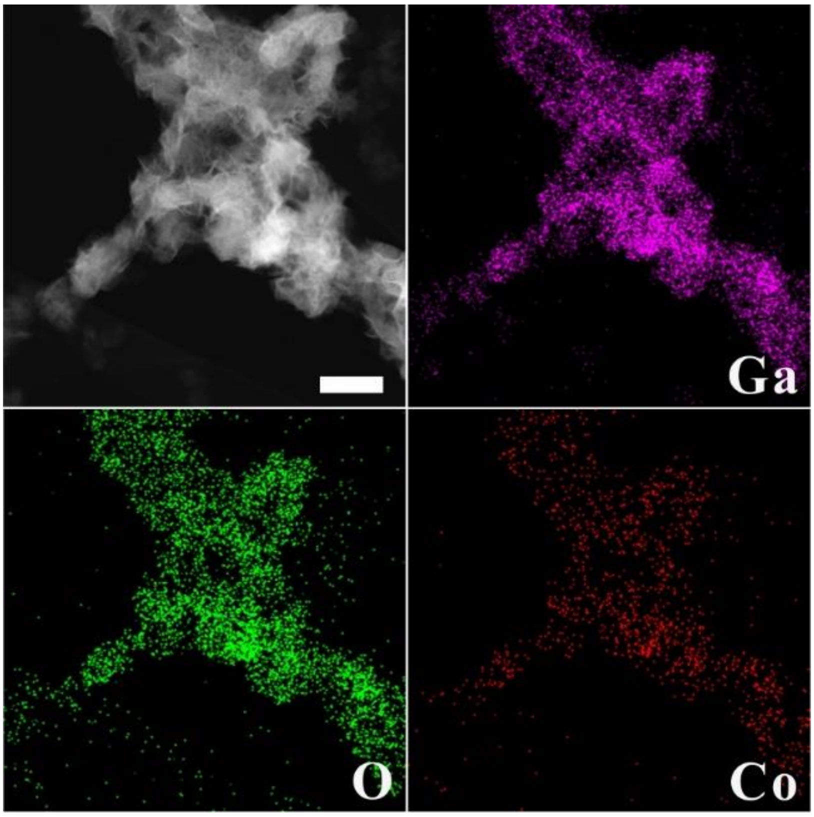

Figure S4 Annular dark-field TEM image and the corresponding elemental mapping images of the Co-Ga_2O_3 nanosheets, in which the scale bar is 200 nm.

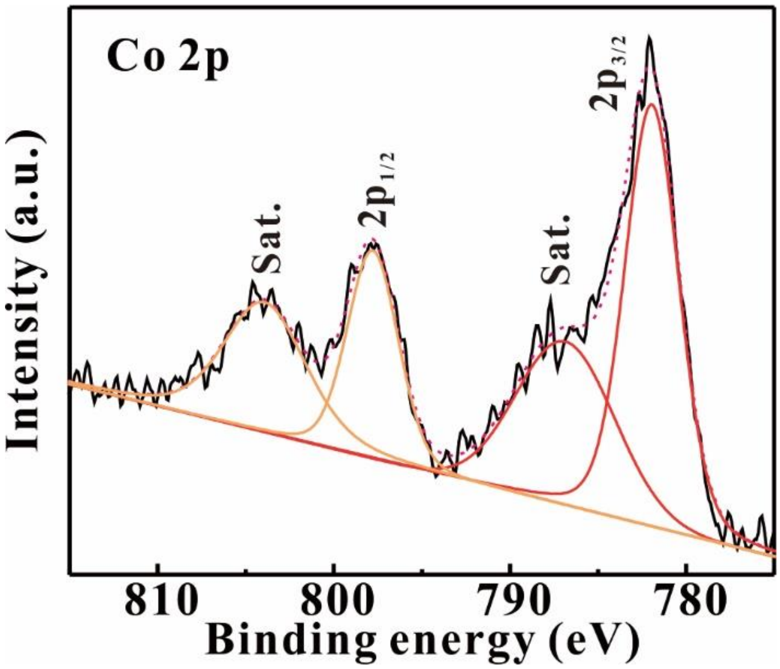

Figure S5 XPS spectra of Co 2p core level for the Co-Ga_2O_3 nanosheets.

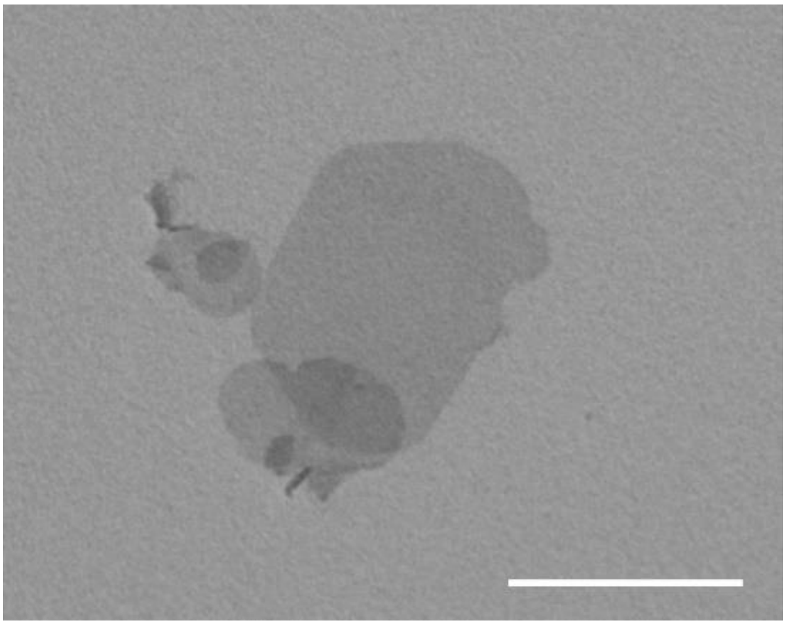

Figure S6 TEM image of the Ga_2O_3 nanosheets, in which the scale bar is 200 nm.

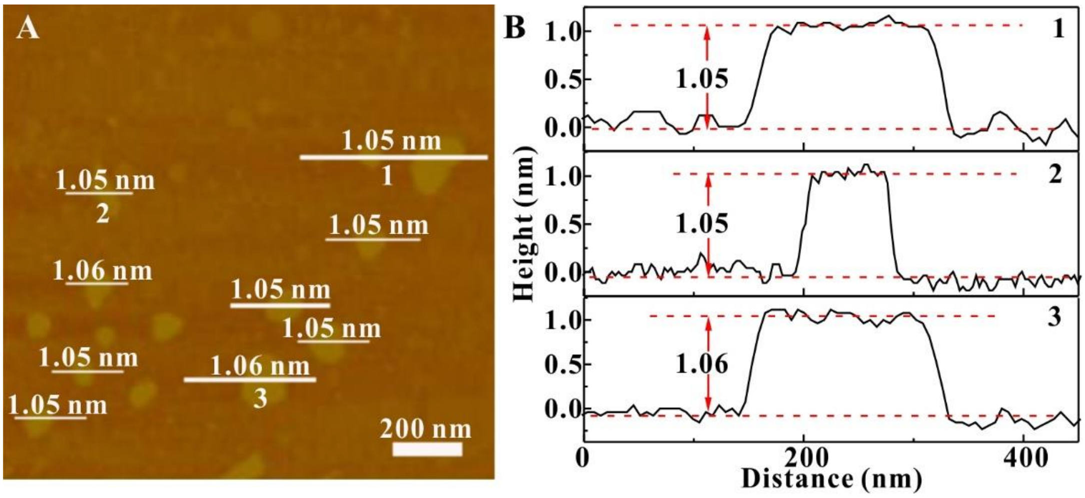

Figure S7 (A) AFM image and (B) the corresponding height profiles of the Ga_2O_3 nanosheets. The number 1, 2 and 3 in (B) correspond to the numbers 1 to 3 in (A), respectively.

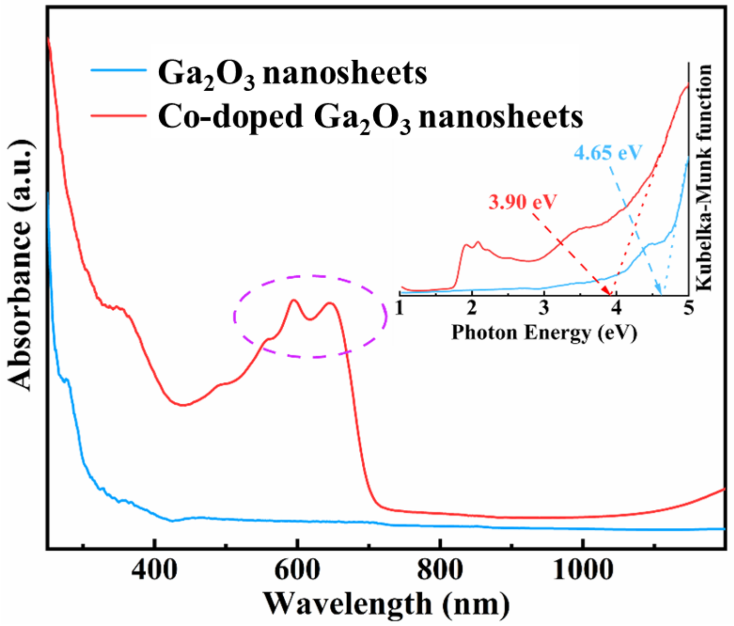

Figure S8 UV−vis diffuse reflectance spectra for the Co-Ga_2O_3 nanosheets and the Ga_2O_3 nanosheets, in which the violet circled area indicated the obviously enhancement of photoabsorption due to the Co-doping. Inset denotes the corresponding Kubelka-Munk plots.

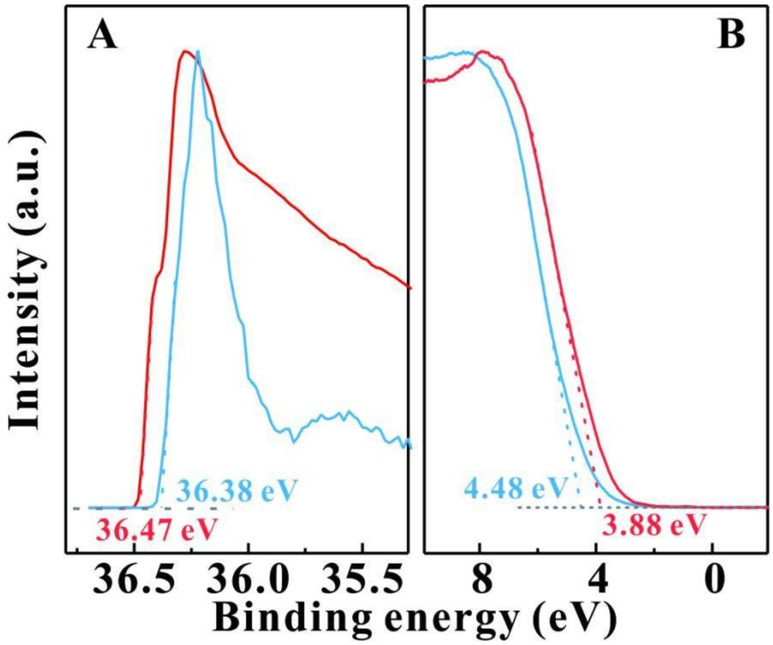

Figure S9 (A) Secondary electron cutoff and (B) valence-band spectra for Co-Ga_2O_3 nanosheets (red) and Ga_2O_3 nanosheets (blue) measured by synchrotron-radiation photoemission spectroscopy spectra.

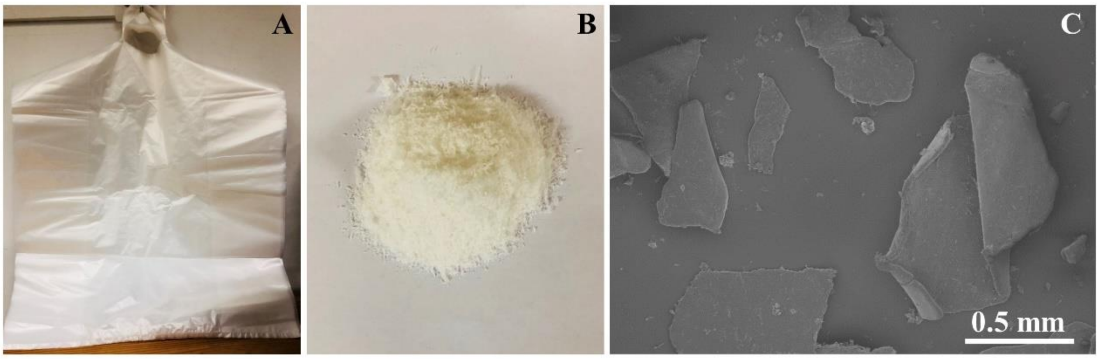

Figure S10 (A) Commercial PE plastic bags and (B) the fine powders obtained by pulverizing the commercial PE plastic bags. (C) The SEM image of the powders of commercial PE plastic bags.

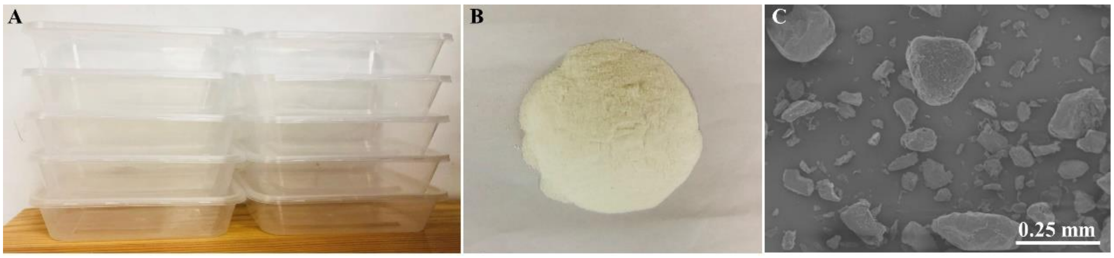

Figure S11 (A) Commercial PP plastic boxes and (B) the fine powders obtained by pulverizing the commercial PP plastic boxes. (C) The SEM image of the powders of commercial PP plastic boxes.

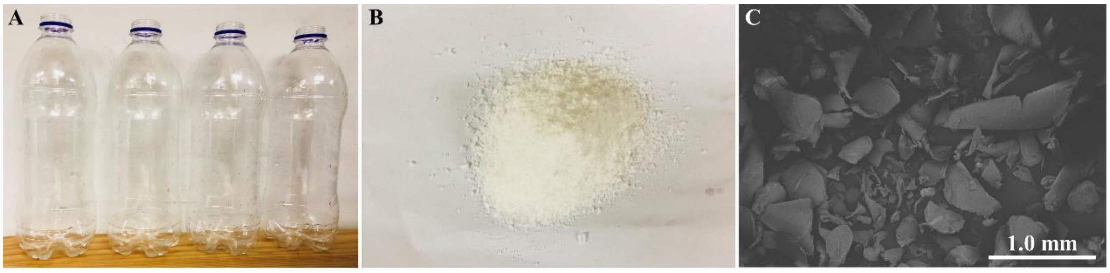

Figure S12 (A) Commercial PET plastic bottles and (B) the fine powders obtained by pulverizing the commercial PET plastic bottles. (C) The SEM image of the powders of commercial PET plastic bottles.

As displayed in Figures S10-S12, commercial plastics including PE plastic bags, PP plastic boxes and PET plastic bottles were initially crushed into fine powders by a pulverizer to ensure their good contact with H_2O. The corresponding SEM images showed that the sizes of most powders are smaller than 5 mm.

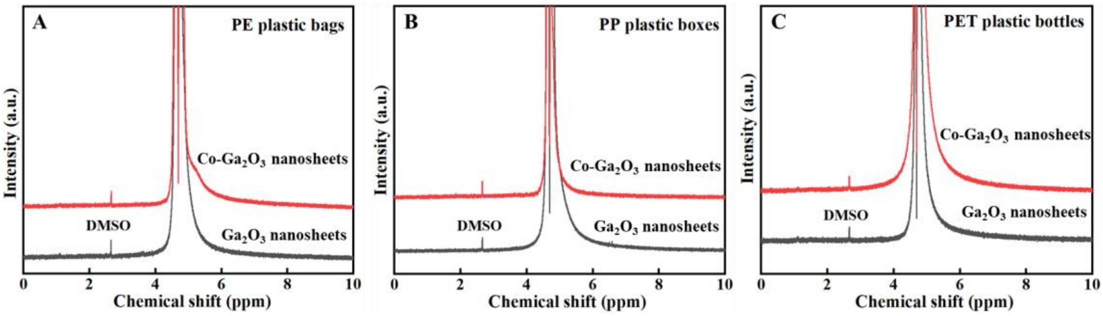

Figure S13 ^{1}H NMR spectra for the products for the photoconversion of (A) PE plastic bags, (B) PP plastic boxes and (C) PET plastic bottles after 24 h irradiation over the Co-Ga_2O_3 nanosheets and Ga_2O_3 nanosheets under mild conditions.

As displayed in Figures S13, there was no detectable liquid product for all these three plastics after 24 h irradiation over the Co-Ga_2O_3 nanosheets and Ga_2O_3 nanosheets under mild conditions.

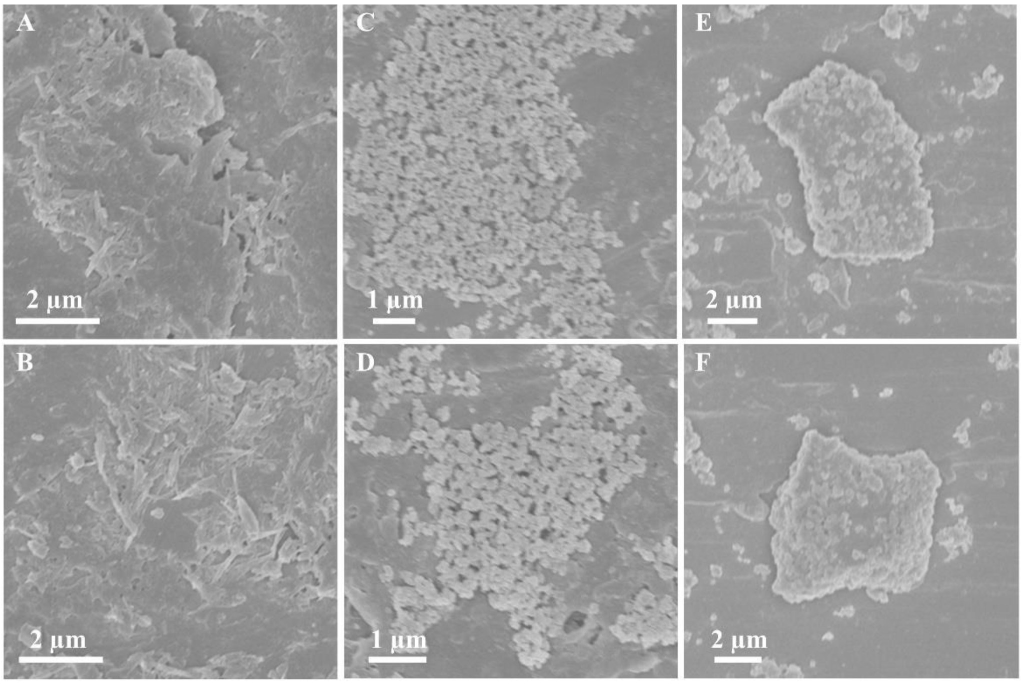

Figure S14 The SEM images of microplastics in the solution after the photoconversion of (A, B) PE plastic bags, (C, D) PP plastic boxes and (E, F) PET plastic bottles after 24 h irradiation over the Co-Ga_2O_3 atomic layers and Ga_2O_3 atomic layers under mild conditions, respectively.

As displayed in Figures S14, a handful of microplastics with smaller size were also detected, which might be produced during the photoconversion processes, conforming to the principle of carbon balance.

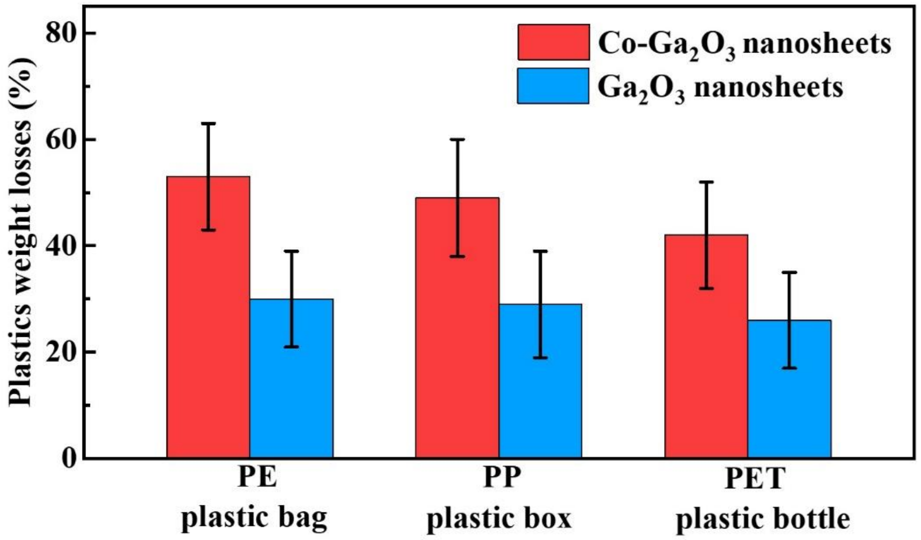

Figure S15 The weight losses of commercial PE plastic bags, PP plastic boxes and PET plastic bottles for the Co-Ga_2O_3 nanosheets and the Ga_2O_3 nanosheets in a simulated sunlight (AM 1.5G, 100 mW/cm^2) at ambient temperature and pressure after 24 h irradiation, respectively. The error bars represent the standard deviations of three independent measurements.

After the photodegradtion processes, the residual plastic powders were collected to calculated their weight losses. To be specific, the plastic powders and photocatalysts were collected by centrifugation after the photoconversion (total residual weight, W1). To lower the deviation, the residual weight of the photocatalysts (W2) were estimated first (when 50 mg photocatalysts were dispersed into 100 mL H_2O, around 46 mg samples could be collected by centrifugation). Then the weight losses of plastics were calculated by the following formula:

$$\text{Plastics weight losses } (\%) = 1 - \frac{\text{W1 - W2}}{\text{initial mass of plastics}} \qquad \text{(Eq. S9)}$$

Considering the losses of the plastics during the centrifugation, the calculated plastics weight losses may still be little high. The weight losses of PE plastic bags powders were 53%, 49% and 42% for the Co-Ga_2O_3 nanosheets, while those were 30%, 29% and 26% for the Ga_2O_3 nanosheets after 24 h irradiation. More importantly, the weight losses of the PE plastic bags, PP plastic boxes and PET plastic bottles were up to ca. 81%, 78% and 72% after 48 h irradiation over the Co-Ga_2O_3 nanosheets.

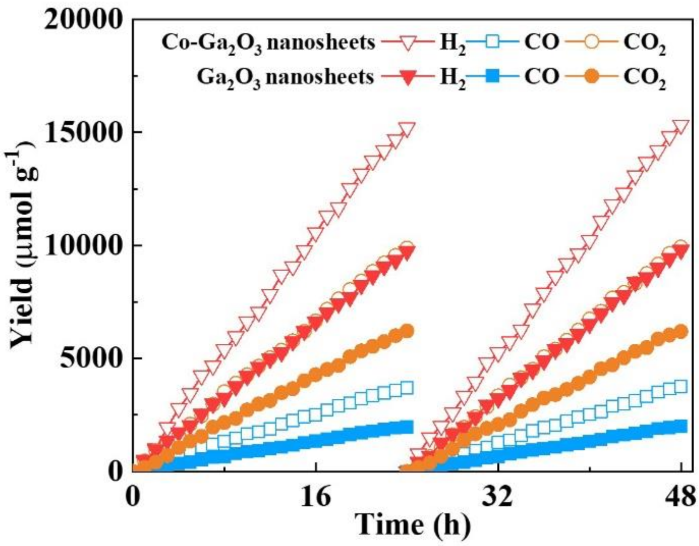

Figure S16 Cycling stability tests: another 100 mg commercial PE plastic bags were added to the system after 24 h irradiation, then the reaction system was vacuum-treated and refilled with high-purity air to reach an atmospheric pressure.

As shown in Figure S16, the formation rates of H_2, CO and CO_2 for the Co-Ga_2O_3 nanosheets did not show any obvious drop upon adding another 100 mg commercial PE plastic bags to the system after 24 h irradiation for the cycling measurements, suggesting their superb photocatalytic stability.

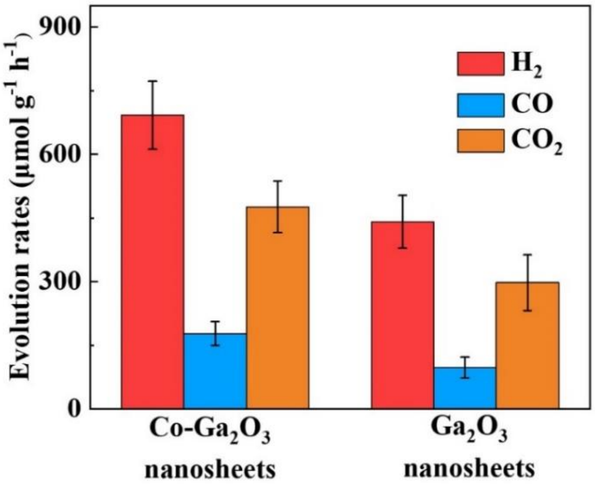

Figure S17 Photoconversion of reagent-grade PE powders over the Co-Ga_2O_3 nanosheets and the Ga_2O_3 nanosheets in a simulated sunlight (AM 1.5G, 100 mW/cm^2) at ambient temperature and pressure: the formation rates of H_2, CO and CO_2. The error bars represent the standard deviations of three independent measurements.

Taking commercial PE powders as example, the Co-Ga_2O_3 nanosheets displayed H_2, CO and CO_2 formation rates of 692.0, 177.8 and 476.4 μmol g^{-1} h^{-1}, while those of the Ga_2O_3 nanosheets were 441.1, 97.6 and 297.7 μmol g^{-1} h^{-1}, respectively.

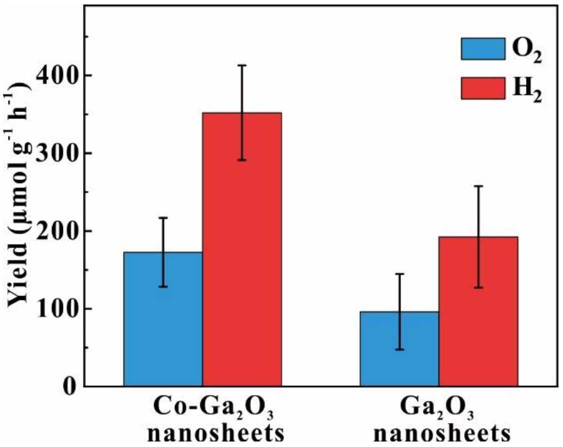

Figure S18 Photocatalytic water-splitting tests for the Co-doped Ga_2O_3 nanosheets and the Ga_2O_3 nanosheets in N_2 atmosphere (no PE powders were added): the formation rates of H_2 and O_2 in 5 h. The error bars represent the standard deviations of three independent measurements.

As shown in Figure S18, H_2O could be directly split into O_2 and H_2 over the Co-doped Ga_2O_3 nanosheets and the Ga_2O_3 nanosheets when no PE powders were added. The formation rates of H_2 and O_2 of the Co-doped Ga_2O_3 nanosheets were 352.0 and 164.9 μmol g^{-1} h^{-1}, while those for the Ga_2O_3 nanosheets were 191.4 and 86.1 μmol g^{-1} h^{-1} respectively.

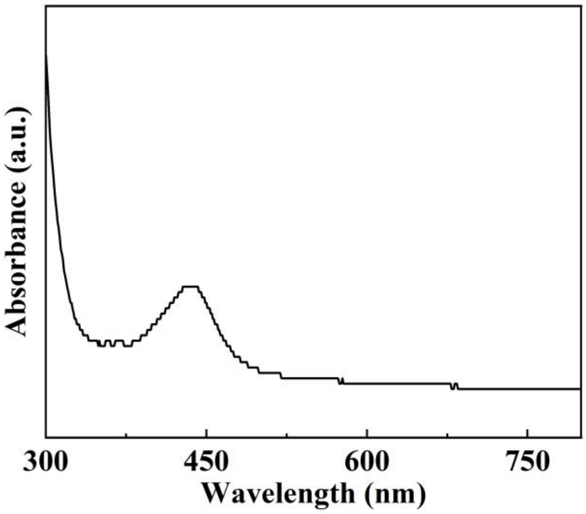

Figure S19 UV–vis absorption spectrum of the reaction solution after the photocatalytic water-splitting tests for 5 h over the Co-Ga_2O_3 nanosheets.

As shown in Figure S19, some amount of H_2O_2 was formed during the water splitting process over the Co-Ga_2O_3 nanosheets.

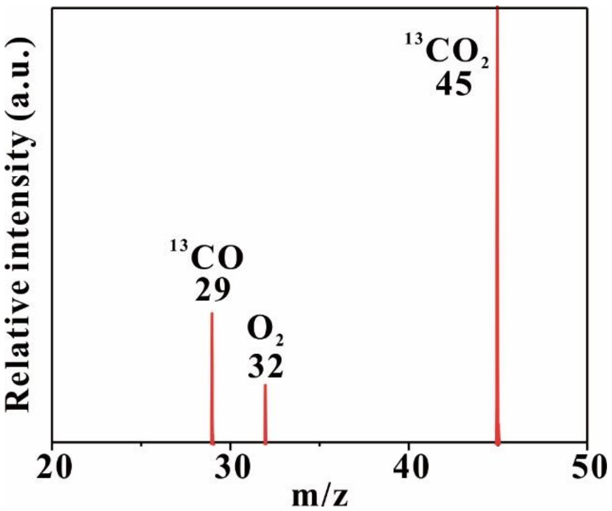

Figure S20 Synchrotron-based vacuum UV photoionization mass spectrometry (SVUV-PIMS) of the gas products after $^{13}CO_2$ photoreduction over the Co-Ga_2O_3 nanosheets at hν = 14.5 eV.

As revealed in Figure S20, ^{13}CO was detected after the CO_2 photoreduction experiments, which clearly showed that CO came from the reduction of CO_2.

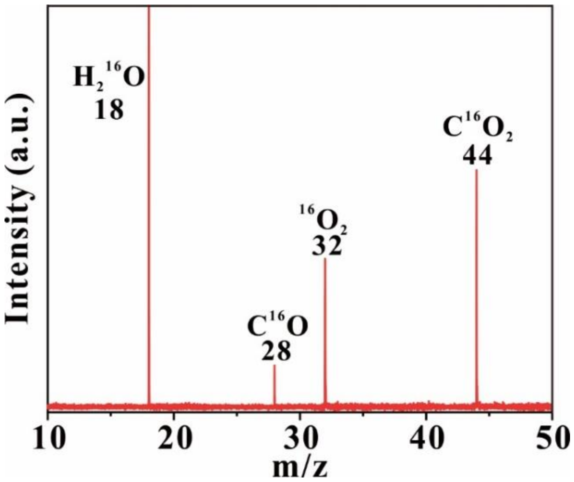

Figure S21 The synchrotron-based vacuum UV photoionization mass spectrometry (SVUV-PIMS) of the gas products after 24 h irradiation: blank controlled experiments for the photoconversion of reagent-grade PE powders over the Co-Ga_2O_3 nanosheets at hν = 14.5 eV. The reaction system was vacuum-treated three times to remove air completely, which was then pumped by high-purity N_2 to reach an atmospheric pressure.

As displayed in Figure S21, upon removing $H_2{}^{18}O$ and $^{18}O_2$ from the reaction system, no $C^{16}O^{18}O$ was detected in blank controlled experiments, further confirming that H_2O and O_2 participated in the oxidation of PE powders into CO_2.

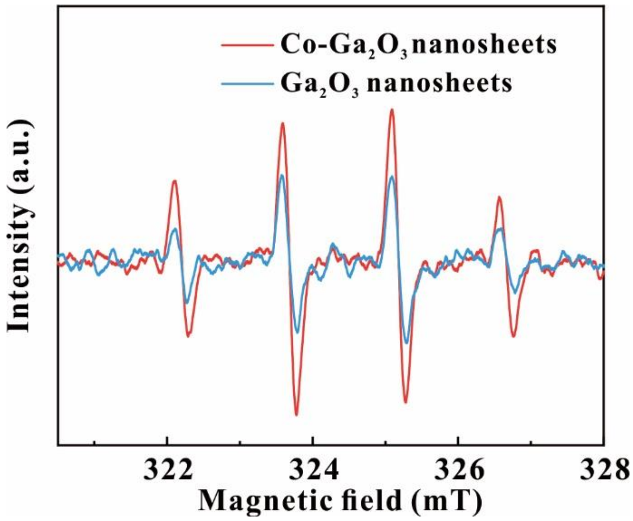

Figure S22 *In situ* ESR spectra during the photoconversion of reagent-grade PE powders over the Co-Ga_2O_3 nanosheets and the Ga_2O_3 nanosheets with H_2O as solvent and DMPO as spin-trapping agent.

As shown in Figure S22, the ESR signals exhibited 1:2:2:1 quartet pattern in water solution, which could be assigned to the ·OH radicals captured by DMPO.

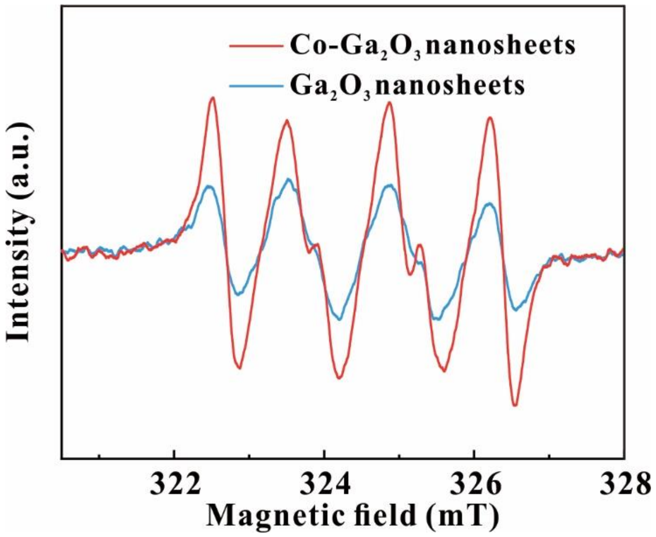

Figure S23 *In situ* ESR spectra during the photoconversion of reagent-grade PE powders over the Co-Ga_2O_3 nanosheets and the Ga_2O_3 nanosheets with methanol as solvent and DMPO as spin-trapping agent.

As displayed in Figure S23, the ESR signals showed quartet pattern with the intensity of nearly 1:1:1:1 in methanol solution, which could be assigned to the superoxide radicals ($O_2^{\cdot-}$) captured by DMPO.

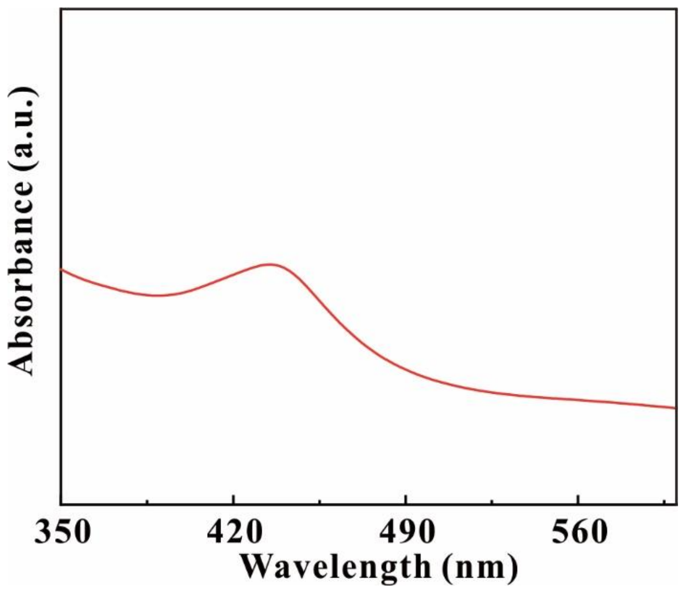

Figure S24 UV–vis absorption spectrum for the reaction solution after 24 h irradiation of the reagent-grade PE photoconversion over the Co-Ga_2O_3 nanosheets.

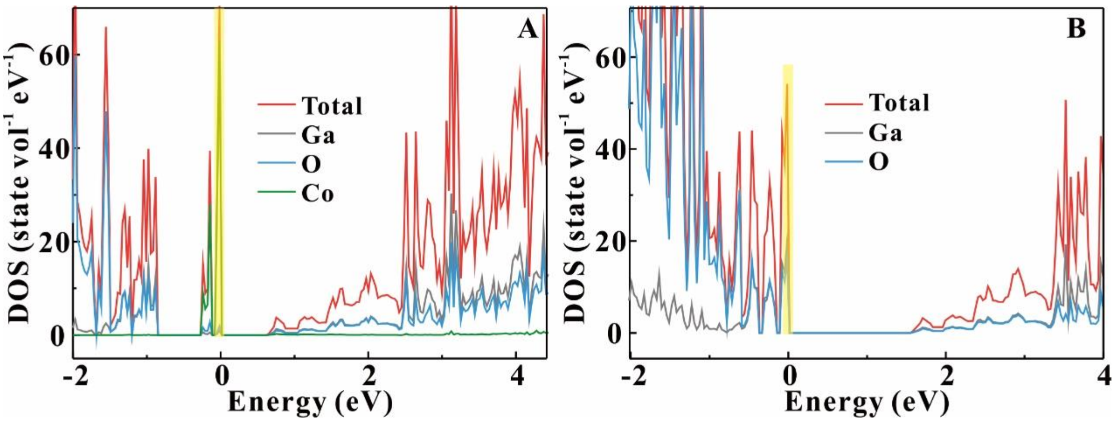

Figure S25 Density of states of (A) the Co-Ga_2O_3 nanosheets and (B) the Ga_2O_3 nanosheets. The yellow bars in A and B represent the valence band edge.

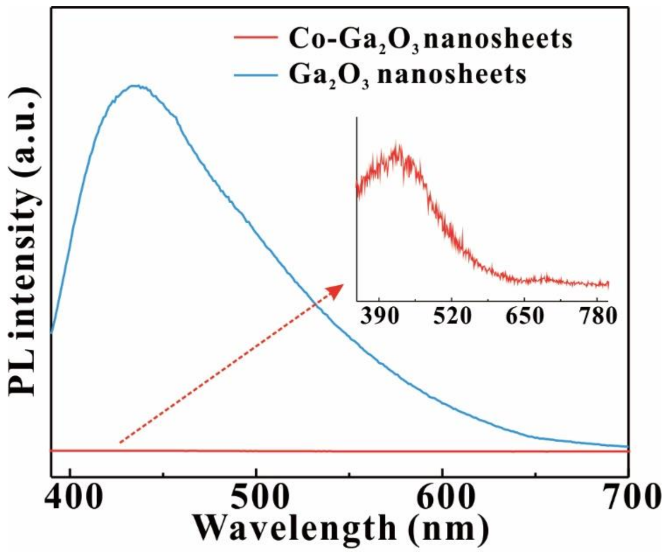

Figure S26 PL spectra for the Co-Ga_2O_3 nanosheets and the Ga_2O_3 nanosheets.

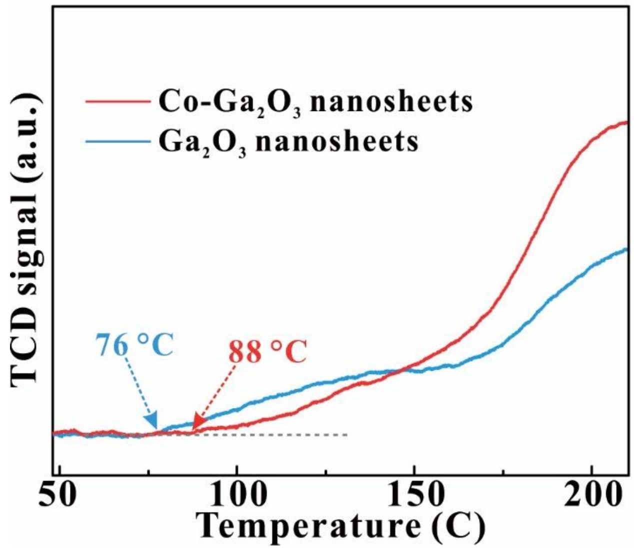

Figure S27 CO_2 temperature programmed desorption (CO_2-TPD) measurements of the Co-Ga_2O_3 nanosheets and the Ga_2O_3 nanosheets.

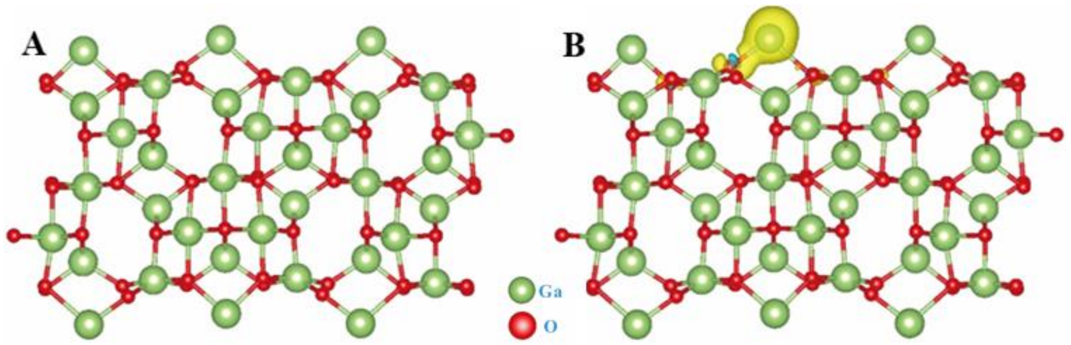

Figure S28 (A) Optimized crystal structure of the Ga_2O_3 nanosheets and (B) the corresponding differential charge density map. The value of isosurfaces is 0.007 eÅ^{-3}, while the yellow and blue contours manifest electron accumulation and depletion, respectively.

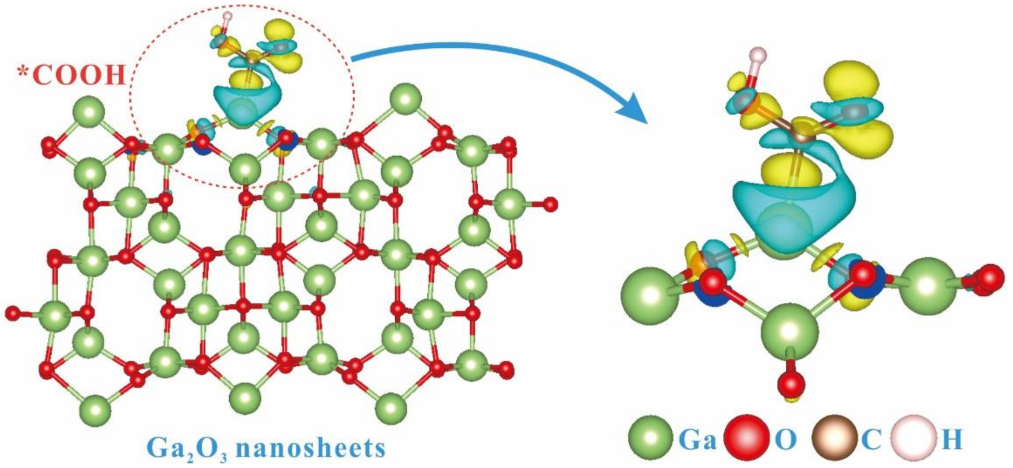

Figure S29 The differential charge density maps of *COOH for the Ga_2O_3 nanosheets, in which the yellow and blue contours manifest electron accumulation and depletion, respectively. The value of isosurfaces is 0.002 eÅ^{-3}.

As shown in Figure S28, the charge of Ga on the Ga_2O_3 nanosheets mainly localize on Ga atoms. The introduction of Co atoms causes the charge redistribution of the neighboring Ga atoms, and the charge of Ga atoms would shift to the Co side (Figure 3D), which helps to stabilize the *COOH and lower its formation energy (Figure S29, Figure 3B, Figure 3E).

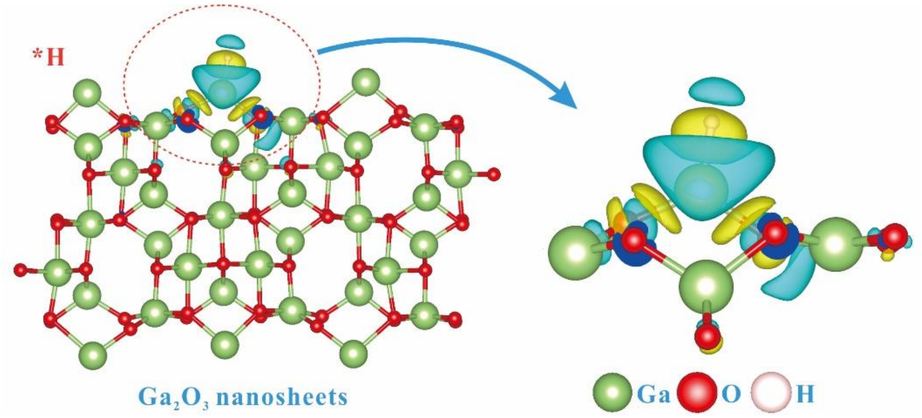

Figure S30 The differential charge density maps of *H for Ga_2O_3 nanosheets, in which the yellow and blue contours manifest electron accumulation and depletion, respectively. The value of isosurfaces is 0.002 eÅ^{-3}.

As shown in Figure S30 and Figure 3C, 3F, the introduction of Co atoms results in the increased charge density around Co atoms that would tend to bond with the *H intermediates and hence lowers their formation energies.

Table S1

The formation rates of H_2, CO and CO_2 during the photoconversion of various commercial plastic products on the Co-Ga_2O_3 nanosheets and the Ga_2O_3 nanosheets with a simulated sunlight (AM 1.5G, 100 mW/cm^2) at ambient temperature and pressure**.**

Photocatalysts	Products (μmol g^{-1} h^{-1})	PE plastic bags	PP plastic boxes	PET plastic bottles
Co-Ga_2O_3 nanosheets	H_2	647.8	603.5	384.2
	CO	158.3	147.2	100.6
	CO_2	419.3	389.1	258.9
Ga_2O_3 nanosheets	H_2	408.0	392.7	218.3
	CO	84.2	75.6	61.8
	CO_2	263.9	252.4	163.4

Table S2

The weight losses of commercial PE plastic bags, PP plastic boxes and PET plastic bottles for the Co-Ga_2O_3 nanosheets and the Ga_2O_3 nanosheets with a simulated sunlight (AM 1.5G, 100 mW/cm^2) at ambient temperature and pressure after 24 h irradiation**.**

Photocatalysts	PE plastic bags	PP plastic boxes	PET plastic bottles
Co-Ga_2O_3 nanosheets	53%	49%	42%
Ga_2O_3 nanosheets	30%	29%	26%

Table S3

Control experiments: the formation rates of H_2, CO and CO_2 during the photoconversion of reagent-grade PE powders on the Co-Ga_2O_3 nanosheets and the Ga_2O_3 nanosheets with a simulated sunlight (AM 1.5G, 100 mW/cm^2) at ambient temperature and pressure when light, photocatalysts or PE powders was removed**.** The "n.d." refers to "not detected".

Photocatalysts	Products (μmol g^{-1} h^{-1})	No light	No photocatalysts	No PE powders
Co-Ga_2O_3 nanosheets	H_2	n.d.	n.d.	329.3
	CO	n.d.	n.d.	n.d.
	CO_2	n.d.	n.d.	n.d.
Ga_2O_3 nanosheets	H_2	n.d.	n.d.	175.5
	CO	n.d.	n.d.	n.d.
	CO_2	n.d.	n.d.	n.d.

Control experiments showed that only H_2 was detected when no PE powders was added in the photocatalytic system, suggesting that H_2 may be came from the reduction of water. No CO and CO_2 were detected upon light, photocatalysts or PE powders was removed, which firmly confirmed CO and CO_2 came from the photodegradation of reagent-grade PE powders by the photocatalysts.

Table S4

Control experiments: the formation rates of H_2, CO and CO_2 during the photoconversion of reagent-grade PE powders on the Co-Ga_2O_3 nanosheets and the Ga_2O_3 nanosheets with a simulated sunlight (AM 1.5G, 100 mW/cm^2) at ambient temperature and pressure when 2 mmol/L $AgNO_3$ solution, or high-purity O_2, or N_2 was used**.** The "n.d." refers to "not detected".

Photocatalysts	Products (μmol g^{-1} h^{-1})	$AgNO_3$	O_2	N_2
Co-Ga_2O_3 nanosheets	H_2	n.d.	673.2	787.7
	CO	n.d.	175.6	76.5
	CO_2	735.6	508.2	214.7
Ga_2O_3 nanosheets	H_2	n.d.	430.8	505.6
	CO	n.d.	94.5	48.3
	CO_2	500.7	312.9	139.8

Table S5

The calculation of carbon balance after the photodegradation of commercial plastic products on Co-Ga_2O_3 nanosheets in simulated sunlight (AM 1.5G, 100 mW/cm^2) at ambient temperature and pressure.

Plastic types	Carbon Losses (mmol)	CO_2+CO (gas, mmol)	CO_2+CO (dissolved, mmol)	Carbon Balance (%)
PE bags	3.79	0.69	2.88	94.2
PP boxes	3.50	0.64	2.53	90.6
PET bottles	2.19	0.43	1.65	95.0

Table S6 The calculation of carbon balance after the photodegradation of commercial plastic products on Ga_2O_3 nanosheets in simulated sunlight (AM 1.5G, 100 mW/cm^2) at ambient temperature and pressure.

Plastic type	Carbon Losses (mmol)	CO_2+CO (gas, mmol)	CO_2+CO (dissolved, mmol)	Carbon Balance (%)
PE bags	2.14	0.42	1.56	92.5
PP boxes	2.07	0.39	1.48	90.3
PET bottles	1.35	0.27	0.99	93.3

Table S7

Free energy and free energy corrections (eV) for species**.**

Species	E_{DFT}	ZPE	TΔS	G
H_2O	-14.21	0.56	0.67	-14.32
H_2	-6.76	0.27	0.40	-6.89
CO_2	-22.98	0.31	0.66	-23.33
CO	-14.79	0.13	0.60	-15.26
*CO		0.22	0.08	
*COOH		0.64	0.15	
*H		0.176	0.02	

Table S8

Free energy (eV) of the Co-Ga_2O_3 nanosheets and the Ga_2O_3 nanosheets with and without intermediates.

Samples	G(*)	G(*COOH)	G(*CO)	G(*H)
Ga_2O_3 nanosheets	-604.62	-629.65	-619.27	-607.19
Co-Ga_2O_3 nanosheets	-606.95	-632.92	-622.62	-609.71

REFERENCES

[1] Kresse G and Furthmuller J. Efficiency of ab-initio total energy calculations for metals and semiconductors using a plane-wave basis set. Comput. *Mater Sci* 1996, **6**: 15-50.

[2] Surendranath Y, Kanan MW and Nocera DG. Mechanistic Studies of the Oxygen Evolution Reaction by a Cobalt-Phosphate Catalyst at Neutral pH. *J Am Chem Soc* 2010, **132**: 16501-16509.

[3] Zhang YK and Yang WT. Comment on "Generalized gradient approximation made simple". *Phys Rev Lett* 1998, **80**: 890-890.
